# Estimating One-Year Risk of Incident Chronic Kidney Disease: Retrospective Development and Validation Study Using Electronic Medical Record Data From the State of Maine

**DOI:** 10.2196/medinform.7954

**Published:** 2017-07-26

**Authors:** Shiying Hao, Tianyun Fu, Qian Wu, Bo Jin, Chunqing Zhu, Zhongkai Hu, Yanting Guo, Yan Zhang, Yunxian Yu, Terry Fouts, Phillip Ng, Devore S Culver, Shaun T Alfreds, Frank Stearns, Karl G Sylvester, Eric Widen, Doff B McElhinney, Xuefeng B Ling

**Affiliations:** ^1^ Department of Epidemiology and Health Statistics School of Public Health, School of Medicine Zhejiang University Hangzhou China; ^2^ Department of Cardiothoracic Surgery Stanford University Stanford, CA United States; ^3^ Clinical and Translational Research Program Betty Irene Moore Children's Heart Center Lucile Packard Children’s Hospital Palo Alto, CA United States; ^4^ HBI Solutions Inc Palo Alto, CA United States; ^5^ Department of Surgery Stanford University Stanford, CA United States; ^6^ China Electric Power Research Institute Beijing China; ^7^ School of Management Zhejiang University Hangzhou China; ^8^ Department of Oncology The First Hospital of Shijiazhuang Shijiazhuang China; ^9^ Empactful Capital San Francisco, CA United States; ^10^ Sequoia Hospital Redwood City, CA United States; ^11^ HealthInfoNet Portland, ME United States

**Keywords:** electronic medical record, chronic kidney disease, risk model, retrospective study

## Abstract

**Background:**

Chronic kidney disease (CKD) is a major public health concern in the United States with high prevalence, growing incidence, and serious adverse outcomes.

**Objective:**

We aimed to develop and validate a model to identify patients at risk of receiving a new diagnosis of CKD (incident CKD) during the next 1 year in a general population.

**Methods:**

The study population consisted of patients who had visited any care facility in the Maine Health Information Exchange network any time between January 1, 2013, and December 31, 2015, and had no history of CKD diagnosis. Two retrospective cohorts of electronic medical records (EMRs) were constructed for model derivation (N=1,310,363) and validation (N=1,430,772). The model was derived using a gradient tree-based boost algorithm to assign a score to each individual that measured the probability of receiving a new diagnosis of CKD from January 1, 2014, to December 31, 2014, based on the preceding 1-year clinical profile. A feature selection process was conducted to reduce the dimension of the data from 14,680 EMR features to 146 as predictors in the final model. Relative risk was calculated by the model to gauge the risk ratio of the individual to population mean of receiving a CKD diagnosis in next 1 year. The model was tested on the validation cohort to predict risk of CKD diagnosis in the period from January 1, 2015, to December 31, 2015, using the preceding 1-year clinical profile.

**Results:**

The final model had a *c*-statistic of 0.871 in the validation cohort. It stratified patients into low-risk (score 0-0.005), intermediate-risk (score 0.005-0.05), and high-risk (score ≥ 0.05) levels. The incidence of CKD in the high-risk patient group was 7.94%, 13.7 times higher than the incidence in the overall cohort (0.58%). Survival analysis showed that patients in the 3 risk categories had significantly different CKD outcomes as a function of time (*P*<.001), indicating an effective classification of patients by the model.

**Conclusions:**

We developed and validated a model that is able to identify patients at high risk of having CKD in the next 1 year by statistically learning from the EMR-based clinical history in the preceding 1 year. Identification of these patients indicates care opportunities such as monitoring and adopting intervention plans that may benefit the quality of care and outcomes in the long term.

## Introduction

Chronic kidney disease (CKD) is a major public health concern in the United States. The National Health and Nutrition Examination Survey (NHANES) reported a prevalence of 15.2% in the general population [1], and it is growing annually, from less than 2% in 2000 to nearly 4.5% in 2008 [2]. The end-stage renal disease prevalence was 2067 per million in the United States in 2014, ranging from 965 to 1754 per million in different health service areas in Maine [3]. CKD is highly associated with other chronic conditions such as diabetes, hypertension, and cardiovascular defects and is associated with poor outcomes and high resource burden [4,5]. Timely recognition and treatment of patients with CKD has been shown to reduce the risk of mortality and complications and slow down disease progression [6-9]. Taken together, these factors highlight a critical need for early detection and intervention to mitigate the impact of CKD.

A barrier to timely recognition and management of CKD is the long clinically silent phase of the disease. Patients with CKD tend to be asymptomatic in the early stage, resulting in generally low awareness of the disease. NHANES reported a self-awareness rate of less than 10% among patients with CKD at stages 1 to 3 and less than 50% at stage 4 [10]. Low awareness of CKD was also found at the provider level, mainly due to poor documentation of the disease and lack of knowledge and education about disease recognition [11-13]. The low awareness at both patient and clinician levels is an impediment to improving the quality of patient care. To increase awareness and thus improve the early recognition from both sides, annual screening with CKD diagnostic testing including serum creatinine and urine albumin testing was recommended for patients at increased risk of CKD, including those with diabetes, hypertension, or family history of kidney disease [14,15]. Yet the existing screening guidelines focus on selected patients rather than the general population, which inevitably tends to ignore a number of CKD patients, especially for those without a history of diabetes or hypertension.

Recent attempts to improve the timely recognition of CKD include identifying risk factors predictive of CKD and combining them to develop a risk score [16-23]. Risk scores stratify individuals based on their probability of having incident CKD or further progression, which can help clinicians to make decisions about intervention. Limitations of those efforts include lack of generalizability across the population, insufficient predictive accuracy, loss to follow-up, and dependence on specific laboratory test results. So far, there is no widely accepted risk assessment model implemented for clinical use in a large, general population.

The widespread use of electronic medical records (EMRs) affords a unique opportunity to understand health care status and improve care management at the population level. The successful use of EMR data to develop risk scores for population stratification has facilitated better patient care for other conditions [24-28]. Enabled by information technology, analysis using EMR data provides a unique perspective on population health tendencies, with large numbers of patients and high dimensional clinical data elements. In this study, we aimed to develop an EMR-based risk model to estimate the probability of receiving an incident diagnosis of CKD within the next 1 year. The model was derived through statistical learning from patients’ prior 1-year clinical history, combined with domain knowledge of risk factors of CKD. The data sources were EMRs collected from 35 hospitals, 34 federally qualified health centers, and more than 400 ambulatory practices in the state of Maine covering more than 1 million patients [27,29]. We aimed to predict patients with newly recognized CKD within the next 1 year. The term “recognized CKD“ included patients having diagnosis codes from the *International Classification of Diseases, Ninth Revision, Clinical Modification* (ICD-9-CM) associated with CKD diagnosis. We hypothesized that the proposed risk model would be able to identify high-risk patients prior to the assignment of a CKD diagnosis code. To our knowledge it is the first study to predict the 1-year risk of being diagnosed with CKD by using EMR data in an all-age, all-disease, and all-payer group general population.

## Methods

### Reporting Method

The study was reported according to the Transparent Reporting of a Multivariable Prediction Model for Individual Prognosis or Diagnosis (TRIPOD) guidelines for a derivation and validation predictive model [30] (Multimedia Appendix 1).

### Ethics Statement

Protected personal health information was removed for the purpose of this research. Because it analyzed de-identified data, this study was exempted from ethics review by the Stanford University Institutional Review Board (October 16, 2014).

### Studied Population and Source of Data

Patient information for this study was extracted from the Health Information Exchange (HIE) dataset administered by HealthInfoNet, an independent nonprofit organization. The dataset contains records of nearly 95% of the population in the state of Maine. Data elements include demographic information, socioeconomic status, laboratory and radiographic tests coded according to Logical Observation Identifier Names and Codes, outpatient medication prescriptions coded according to the National Drug Code, and primary and secondary diagnoses and procedures which are coded using ICD-9-CM. Missing data handling is described in Multimedia Appendix 2. The study included patients who visited any care facility in the Maine HIE network any time from January 1, 2013, through December 31, 2015. Patients who died or had a history of CKD diagnosis at any time between 2009 (the first time deployment of any EMR system in the state of Maine) and the time point of prediction or a history of treatment or diagnosis for end-stage renal disease were excluded from the study.

### Outcome Definition

In this study, a CKD case was defined as having an ICD-9-CM diagnosis code of CKD assigned during any visit during the next 1 year, which refers to the period from January 1, 2014, to December 31, 2014, in the derivation cohort and from January 1, 2015, to December 31, 2015, in the validation cohort. A full list of ICD-9-CM codes of CKD was shown in Table m.1 in the 2015 Annual Data Report of the United State Renal Data System [3]. All cases of CKD, including those specified as stages 1 to 5 as well as those with unspecified stages, were included as study cases. The validity of ICD codes of CKD was reported in previous reports [3,31].

**Figure 1:**
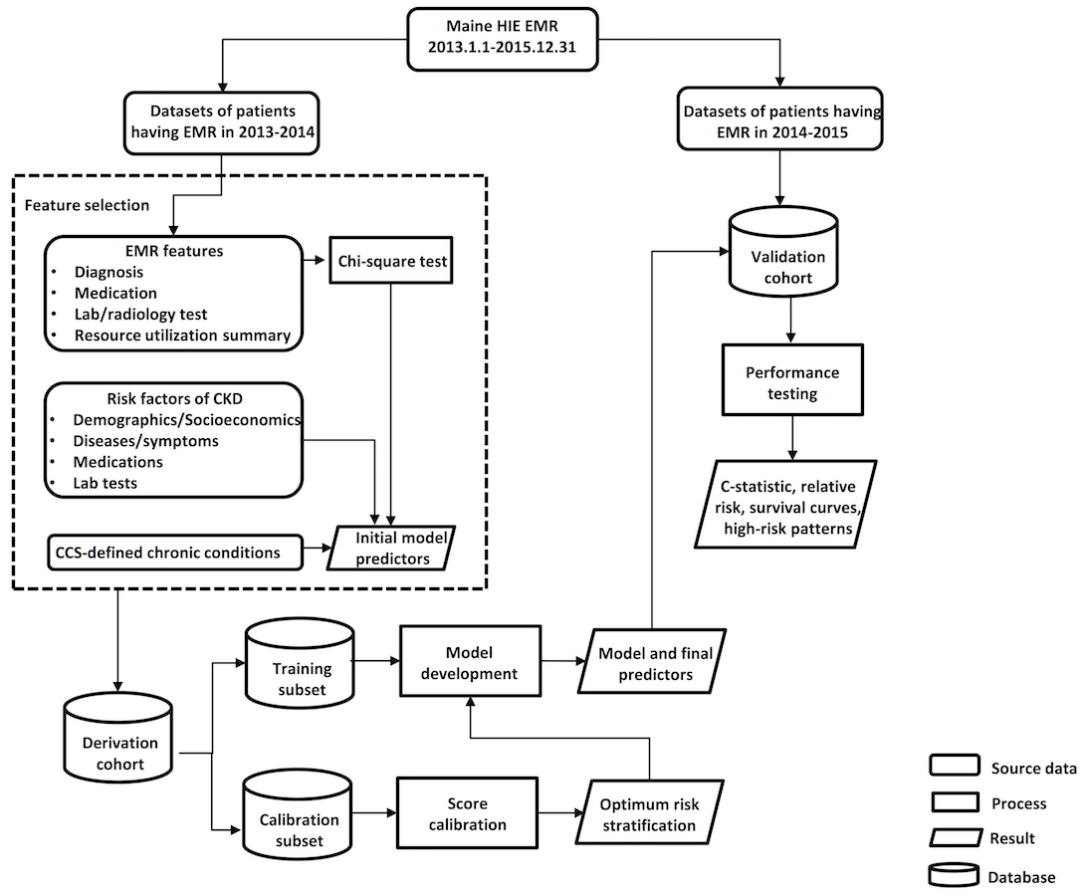
Flow chart of study. Study population was split into two parts based on time frames of electronic medical records (2013-2014 for derivation and 2014-2015 for validation).

**Figure 2:**
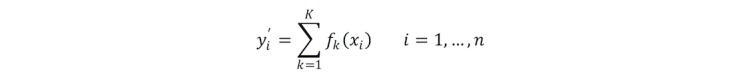
Formula of a tree ensemble model developed with the training subset.

**Figure 3:**
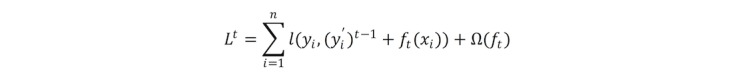
Sum of the loss function and the overfitting control term at the t iteration.

### Feature Selection

A workflow chart for the study is shown in Figure 1. To improve computational efficiency, a feature selection process was carried out to determine the features that would go into the model prior to the derivation phase. The selection process was divided into 2 stages: literature review and variance analysis. Features recognized to have an association with CKD in previous literature were extracted as risk factors. These factors included demographics, chronic disease history, abnormal laboratory test results, and medication prescriptions. Chronic disease history included primary or secondary diagnosis. Medication prescriptions referred to the number of prescriptions for a particular medicine during the past 1 year. Laboratory test results were labeled as abnormal or normal according to thresholds provided by each facility participating in the HIE network. The rest of the features were screened by chi-square test to filter out those not significantly associated with CKD outcome (*P*>.05). The target of this process was to exclude features having low discriminatory power. For example, features that were 0, No, or NA in most of the patient records would probably be removed.

### Derivation Phase

The derivation cohort was divided into 2 subsets for training and calibration purposes. An initial model was derived with the training subset. The model input was the selected features that profiled the preceding 1-year clinical history from January 1, 2013, to December 31, 2013, and the model output was set to either 1 or 0 to indicate whether or not a patient was diagnosed with CKD during the period from January 1, 2014, to December 31, 2014.

A gradient tree-based boosting algorithm was used to develop the model [32]. The idea of the algorithm is to approach the output by an ensemble of classification trees. Assume the training subset had *n* samples (*x*_i_, *y*_i_, *i*=1,…, *n*), a tree ensemble model developed with the training subset can be written according to the formula in Figure 2, where *f* (*x*) is the predictive function of a tree and *K* is the maximum number of trees in algorithm (*K*=500 in this study). Overfitting was avoided by adding a term to penalize the complexity of the algorithm. Parameters were chosen to minimize the sum of loss function and the overfitting control term. See Figure 3 for the sum term at the *t* iteration, where *l* is the loss function, (y_i_’)^t-1^ is the predictive value at the *t*-1 iteration, and *Ω* is the term that controls overfitting. *Ω* is a function of the number of trees and weights of each tree in the algorithm.

An approximate greedy algorithm was used as a splitting method to grow trees. Features on each node were sorted to propose a couple of candidates at percentiles. Splitting points were chosen to optimize purity at the next level. In this study, the maximum depth of each tree was set to 5. Each node was assigned with an estimated value. The final predictive estimate was summed for individual trees.

A calibration subset was used to convert predictive estimates of the model developed with the training subset to a measure of positive predictive values (PPVs), which provided a universal, standardized risk measure. PPV for each predictive estimate y’ was calculated as the proportion of incident CKD events in a subset of samples having predictive estimates higher than y’.

In this way, all the predictive estimates were mapped to the calculated PPVs. The PPVs were defined as scores that described the probability of having a new diagnosis of CKD within the next 1 year. We grouped all patients into 3 categories: low risk, intermediate risk, and high risk, based on the scores.

The scores after calibration were converted to relative risks. The relative risk of each individual was calculated by dividing the score of the individual by the mean score of all patients in the cohort. The relative risk measured the ratio of the probability of having CKD to the baseline. The higher the relative risk, the higher the probability of receiving a diagnosis of CKD in the next 1 year.

### Validation Phase

A validation cohort of patients with clinical history from January 1, 2014, to December 31, 2014, was assembled to test the model performance on predicting the risk of CKD from January 1, 2015, to December 31, 2015. Predicted score and relative risk to the baseline were calculated for each patient. The *c*-statistic, relative risk distribution, and incidence of CKD diagnosis in each risk category were estimated to assess the performance of the model on the validation phase. The performance of the model was also evaluated in subgroups of patients using receiver operating characteristic (ROC) curves and *c*-statistics. Characteristics and clinical patterns of patients in each risk category were compared. Model errors were described by false positives (labeling a patient with no CKD in next 1 year as high risk) and false negatives (labeling a patient with CKD in next 1 year as low or intermediate risk), and clinical patterns of these patients were discussed.

Survival analysis was performed to track the timing of CKD diagnosis in different risk categories. Kaplan-Meier curves were plotted separately for each risk category to compare the probabilities of being diagnosed of CKD at the same time point. The analysis was not censored. A Kruskal-Wallis test was performed to compare the curves between the 3 risk categories. A temporal comparison of the CKD prediction date (ie, the time point when a high-risk patient was identified by the model) and CKD recognized date (ie, the time point when the patient was assigned ICD-9-CM diagnosis codes of CKD) was performed to evaluate the predictive power of the model in the time domain. All analyses were performed using R software (The R Foundation).

## Results

### Study Cohort

The final cohort included 1,310,363 patients for model derivation, 7448 of whom received a new CKD diagnosis in the next 1 year (from January 1, 2014, to December 31, 2014) and 1,430,772 patients for model validation, 8299 of whom had CKD diagnosed in the next 1 year (from January 1, 2015, to December 31, 2015). A cohort construction diagram is shown in Multimedia Appendix 3.

**Table 1:** Baseline characteristics.

Characteristic		Derivation cohort N=1,310,363 n (%)	Validation cohort N=1,430,772 n (%)
**Age (years)**			
	≥65	269,355 (20.56)	299,893 (20.96)
	50-65	288,645 (22.03)	312,456 (21.83)
	40-50	163,792 (12.50)	172,877 (12.08)
	<40	588,571 (44.92)	645,546 (45.12)
Female		690,714 (52.71)	748,867 (52.34)
**Race**			
	White	1,090,046 (83.19)	1,194,478 (83.48)
	Black	18,233 (1.39)	21,770 (1.52)
	Asia	9,082 (0.69)	10,677 (0.75)
	Other^a^/unknown^b^	193,002 (14.73)	203,847 (14.25)
Diabetes		54,366 (4.15)	60,631 (4.24)
Hypertension		121,413 (9.27)	133,328 (9.32)
Heart disease		49,684 (3.79)	52,780 (3.69)
Obesity		37,734 (2.88)	40,765 (2.85)

^a^Other refers to patients labeled as other race, multirace, or mixed.

^b^Unknown refers to patients labeled as unknown, undetermined, not applicable, or declined to answer.

### Baseline Characteristics

The baseline characteristics of patients in derivation and validation cohorts are shown in Table 1. Both cohorts exhibited similar patterns of demographics and clinical conditions. The study involved patients of all ages and was gender balanced. In both cohorts, elderly patients (age ≥65 years) composed around 21% of the cohort, while young adults (<40 years) made up around 45% of the total; 18% of patients were pediatric (<18 years). The majority of patients were white. A history of diabetes or hypertension, 2 well-established risk factors of CKD, was present in approximately 4% and 9%, respectively, of the cohorts. Heart disease and obesity were present in almost 4% and 3%, respectively, in the cohorts.

### Feature Selection

There are 14,680 features to profile each patient’s clinical history in HIE dataset. The literature review identified a total of 153 clinical features as conventional risk factors of CKD, including 10 demographic features, 11 socioeconomic characteristics, 46 diagnostic diseases and conditions, 30 laboratory tests, and 56 medications. In parallel, 399 clinical features were selected after screening by chi-square test. These features, plus 184 chronic conditions identified by Clinical Classifications Software for classifying diagnoses and procedures into clinically meaningful categories (Healthcare Cost and Utilization Project, US Agency for Healthcare Research and Quality), constituted a set of 736 features for model derivation (Multimedia Appendix 4). The derivation process identified 146 features with non-zero weight as the final predictors of the model, including 6 demographic features, 2 socioeconomic characteristics, 36 diagnostic diseases and conditions, 17 laboratory tests, 78 medication prescriptions, and 7 utilization variables (Multimedia Appendix 5). The top 50 features and their weights and odds ratios are listed in Multimedia Appendix 6. The following features played an important role in the model: age; history of diabetes, renal diseases, and heart diseases; history of diabetes and blood pressure medications; and health care resource utilization including length of stay in the hospital, total number of medications, and total number of laboratory tests with abnormal results.

### Derivation Phase

We grouped all patients into 3 categories: low risk (score < 0.005), intermediate risk (score 0.005-0.05), and high risk (score ≥ 0.05). Model outcomes in the derivation phase are shown in Table 2. The model had a *c*-statistic of 0.916 in the derivation cohort. Patients diagnosed with CKD in the next 1 year (n=7448) had a median relative risk of 12.5, meaning that the model predicted these patients to have a probability of having CKD 12.5 times more than the baseline. Of these patients, 16.22% (1208/7448) were classified as low risk, 21.17% (1577/7448) as intermediate risk, and 62.61% (4663/7448) as high risk. The percentage of CKD cases and relative risk had a monotonic increase from low-risk (0.10%, 0.017) to high-risk categories (11.82%, 25.4).

**Table 2:** Comparison of the model outcome in derivation and validation cohorts.

Outcome		Derivation cohort N=1,310,363	Validation cohort N=1,430,772
Diagnosed with CKD^a^in the next 1 year, n (%)		7448 (0.57)	8299 (0.58)
**Risk score model**			
	Baseline score, mean (SD)	0.0050 (0.034)	0.0044 (0.018)
	Baseline score for those diagnosed with CKD in the next 1 year, median (1st quartile, 3rd quartile)	0.063 (0.013, 0.29)	0.049 (0.0079, 0.092)
	Relative risk^b^for those diagnosed with CKD in the next 1 year, median (1st quartile, 3rd quartile)	12.5 (2.6, 57.3)	11.1 (1.8, 21.0)
	CKD diagnosis by risk category: low/intermediate/high	1208/1577/4663	1778/2334/4177
**Percent incidence of CKD diagnosis (95% CI)**			
	Low (score 0-0.005)	0.10 (0-0.30)	0.14 (0-0.45)
	Intermediate (score 0.005-0.05)	1.73 (1.15-2.60)	2.10 (1.10-2.90)
	High (score ≥ 0.05)	11.82 (10.10-13.80)	7.94 (6.50-10.10)
**Relative risk to the population baseline (95% CI)**			
	Low (score 0-0.005)	0.017 (0.012-0.023)	0.011 (0.0067-0.017)
	Intermediate (score 0.005-0.05)	3.2 (3.0-3.3)	4.1 (3.9-4.2)
	High (score ≥ 0.05)	25.4 (23.9-27.2)	18.3 (17.8-19.0)

^a^CKD: chronic kidney disease.

^a^Relative risk of each patient was defined as the ratio of the risk score of the patient to the baseline score (ie, the mean risk score of total population).

### Validation Phase

The performance of the model was slightly reduced in the validation cohort, with a *c*-statistic of 0.871, but had similar results (Table 2). The median relative risk of patients diagnosed with CKD in the next 1 year (n=8299) was 11.1, and 50.33% (4177/8299) of these patients were labeled as high risk. The total numbers of low-, intermediate-, and high-risk patients were 1,266,893, 111,195, and 52,594, respectively, 0.14%, 2.10%, and 7.94%, respectively, of whom had a diagnosis of CKD within the next 1 year.

The cutoff of the high-risk patients (score ≥ 0.05) gave a sensitivity of 62.61% (95% CI 61.50%-63.71%) and a specificity of 97.33% (95% CI 97.30%-97.36%) in the derivation cohort and sensitivity of 50.33% (95% CI 49.25%-51.41%) and a specificity of 96.60% (95% CI 96.57%-96.63%) in the validation cohort. A 2-by-2 contingency table is shown in Multimedia Appendix 7. A list of sensitivities, specificities, and PPVs as a function of cutoffs is shown in Multimedia Appendix 8. Reduction of the specificity from 96.60% to 87.88% will increase the sensitivity from 50.33% to 80.33%, but it will also reduce PPV from 7.94% to 3.72%.

**Figure 4:**
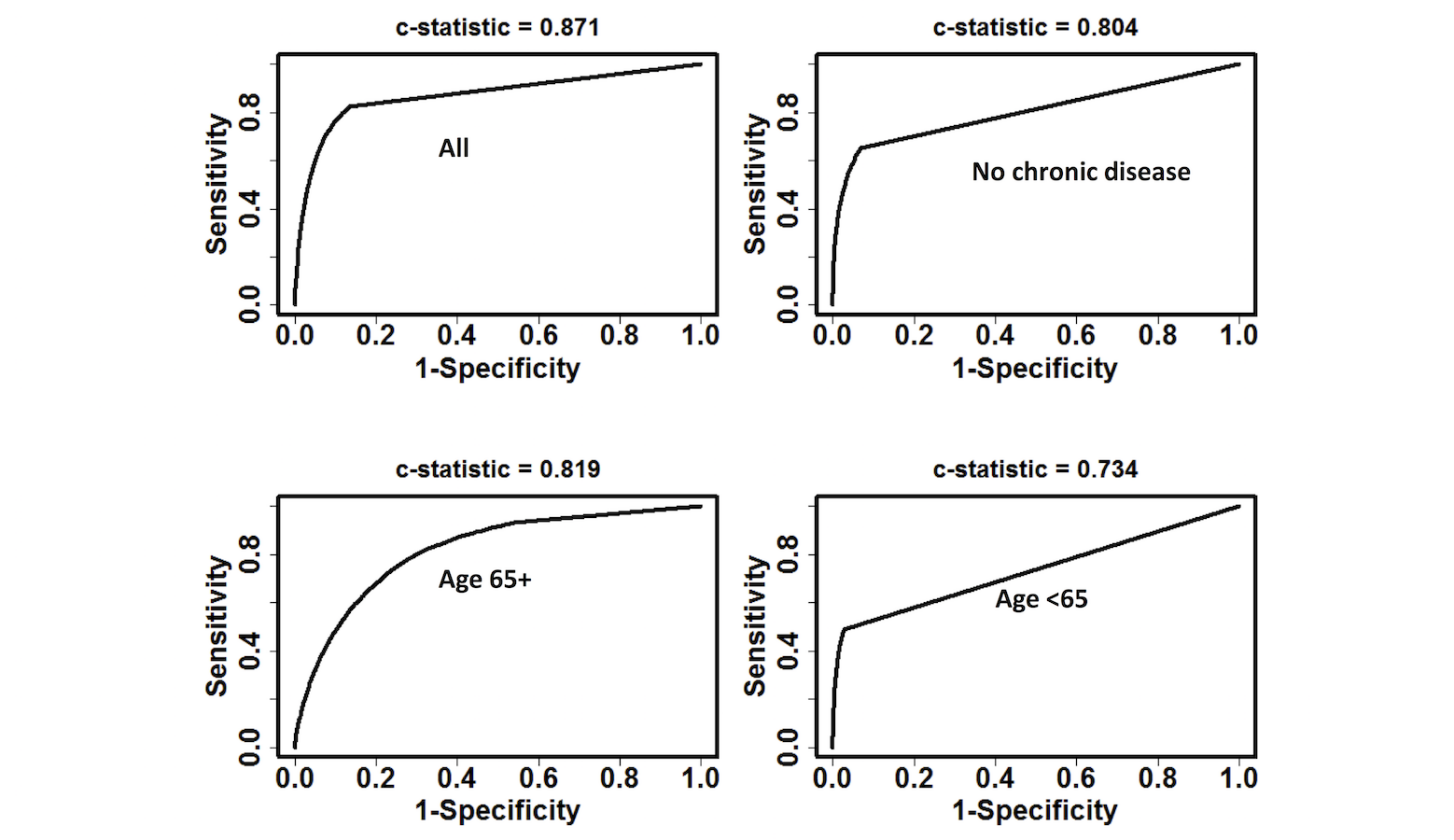
Receiver operating characteristic curves and c-statistics of the model prediction.

As shown in Figure 4, the model had effective discriminatory power within patient subgroups. *C*-statistics for patients with no chronic disease history (741,703/1,430,772, 51.84%), those aged ≥65 years (280,787/1,430,772, 19.62%), and those <65 years (1,149,985/1,430,772, 80.38%) were 0.804, 0.819, and 0.734, respectively.

**Table 3:** Clinical patterns of patients by risk categories in the validation cohort.

Characteristic		Low risk N=1,266,983	Intermediate risk N=111,195	High risk N=52,594
Age, years, median (1st quartile, 3rd quartile)		39 (20, 56)	75 (68, 82)	79 (71, 85)
Female, n (%)		667,440 (52.68)	55,717 (50.11)	25,710 (48.88)
**Race, n (%)**				
	White	1,031,954 (81.45)	110,303 (99.20)	52,221 (99.29)
	Black	21,151 (1.67)	424 (0.38)	195 (0.37)
	Asian	10,332 (0.82)	253 (0.23)	92 (0.17)
	Other/unknown	203,546 (16.07)	215 (0.19)	86 (0.16)
Diabetes, n (%)		22,025 (1.74)	19,271 (17.33)	19,335 (36.76)
Hypertension, n (%)		60,970 (4.81)	39,564 (35.58)	32,794 (62.35)
Heart disease, n (%)		17,388 (1.37)	16,156 (14.53)	19,236 (36.57)
Obesity, n (%)		29,308 (2.31)	6686 (6.01)	4771 (9.07)
Blood pressure medication, n (%)		64,974 (5.13)	42,096 (37.86)	34,183 (64.99)
Diabetes medication, n (%)		26,533 (2.09)	17,045 (15.33)	15,553 (29.57)
Abnormal diabetes test, n (%)		575 (0.05)	388 (0.35)	618 (1.18)
Abnormal urine albumin-to-creatinine ratio, n (%)		155 (0.01)	90 (0.08)	171 (0.33)
Total costs, median (1st quartile, 3rd quartile)		170 (0, 925)	850 (170, 2455)	1700 (510, 4530)
Outpatient visits, median (1st quartile, 3rd quartile)		1 (0, 3)	4 (1, 8)	8 (4, 15)
Total counts of medications, median (1st quartile, 3rd quartile)		0 (0, 3)	7 (0, 31)	32 (7, 75)
Total counts of laboratory tests, median (1st quartile, 3rd quartile)		0 (0, 0)	0 (0, 29)	6 (0, 81)

**Figure 5:**
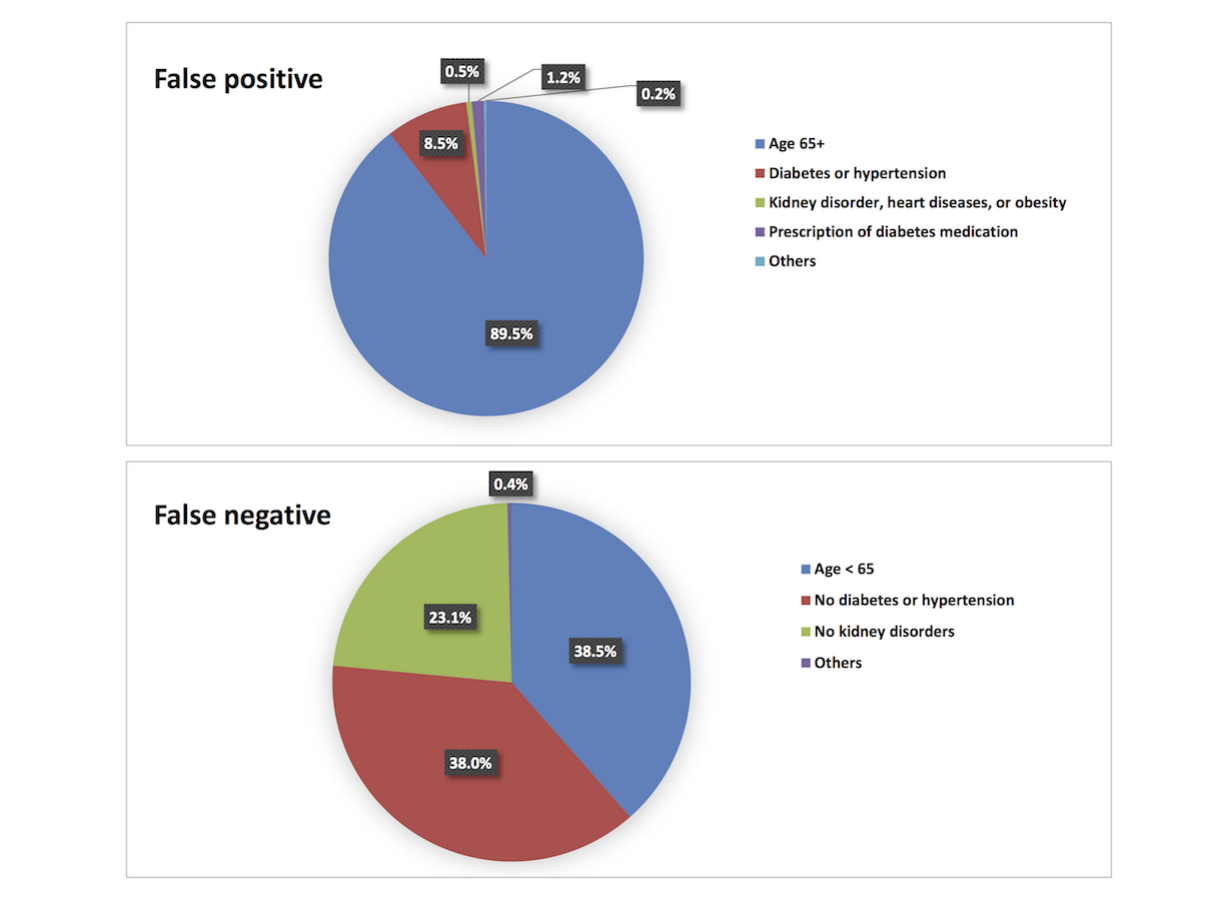
Distribution of false positive patients (top) and false negative patients (bottom) in validation cohort.

Clinical patterns were compared among the low-, intermediate-, and high-risk categories in the validation cohort (Table 3). There was a significant difference (*P*<.001) in age distribution between low- and high-risk patients: 99.86% (644,610/645,546) of young adults (age <40 years) were classified as low risk, while 87.80% (46,175/52,594) of the high-risk patients were ≥65 years of age. Patients in the high-risk category featured more serious comorbidities and more resource consumption. Among high-risk patients, a history of diabetes, hypertension, heart diseases, and obesity was present in 36.76%, 62.35%, 36.57%, and 9.07%, respectively, much higher than in the overall cohort (4.24%, 9.32%, 3.69%, and 2.85%). High-risk patients also utilized the largest amount of resources in terms of total number of outpatient visits (median 8), medications (median 32), and laboratory tests (median 6) over the last 1 year, resulting in the highest annual costs (median $1700) among all 3 risk categories. The model tends to aggregate heavy users of health care resources and those with traditional risk factors of CKD (age, diabetes, and hypertension) into the high-risk category.

### False Positives and False Negatives

The distribution of false positives and false negatives in the validation cohort is shown in Figure 5. Of false positives, 89.53% (43,346/48,417) were patients ≥65 years of age; 8.45% (4092/48,417) were <65 years but with diagnosis of hypertension and/or diabetes; 0.55% (265/48,417) did not have diabetes or hypertension but had kidney disorders, heart diseases, or obesity; and 1.25% (603/48,417) were prescribed medications for diabetes or hypertension. Of the other 111 false positive patients, 110 had at least 1 medication prescription or 1 abnormal laboratory test result during the preceding year. Conversely, there were 4122 false negatives, patients with CKD in next 1 year who were missed by the model. Among these, 38.50% (1587/4122) were <65 years of age, 37.99% (1566/4122) were ≥65 years old but had no history of diabetes or hypertension, and 23.12% (953/4122) had diabetes or hypertension but no kidney disorder.

### Temporal Analysis

**Figure 6:**
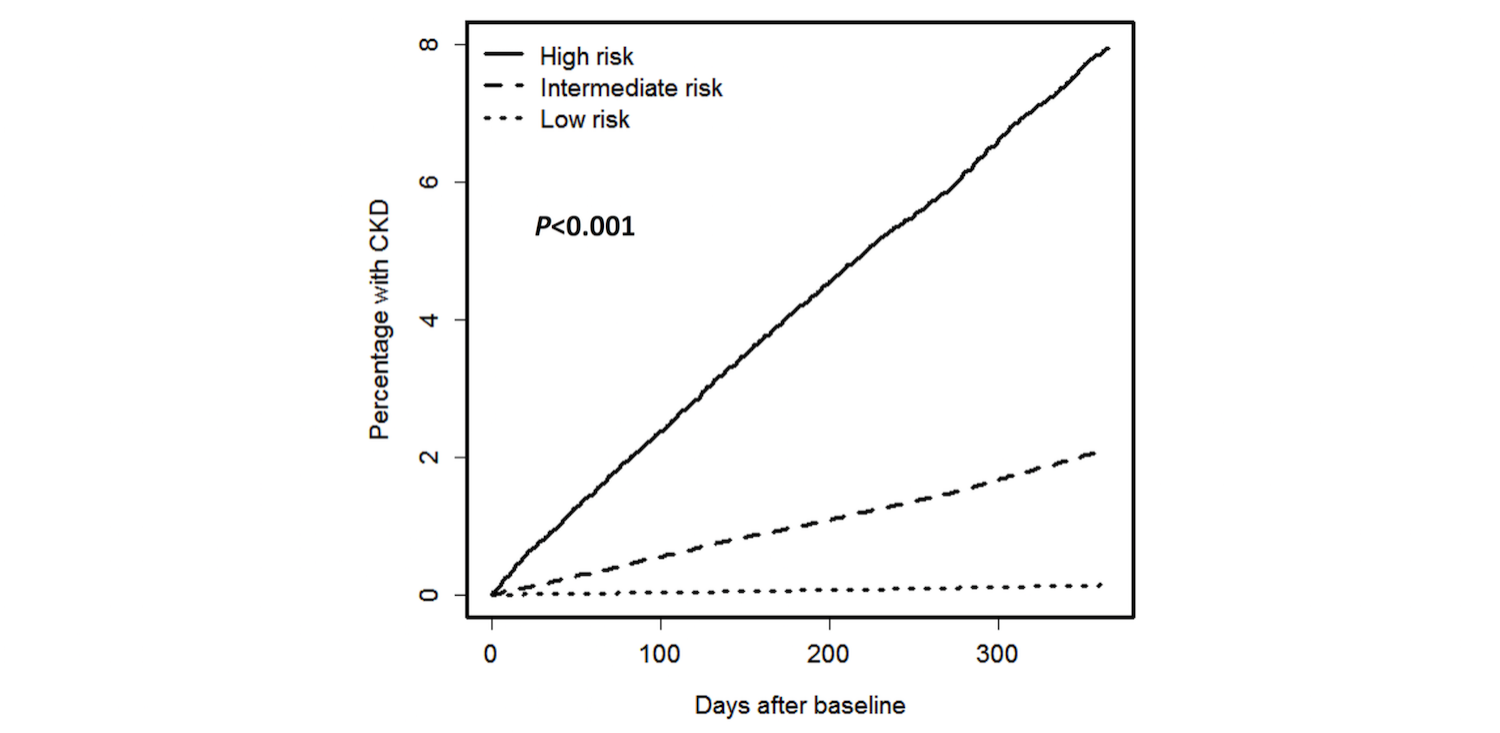
Kaplan-Meier progression to chronic kidney disease for patients in low-, intermediate-, and high-risk categories of validation cohort.

Kaplan-Meier analysis was performed to estimate freedom from a new CKD diagnosis for patients in the 3 risk categories in the validation cohort (Figure 6). Significant differences (*P*<.001) were demonstrated between the risk categories.

**Figure 7:**
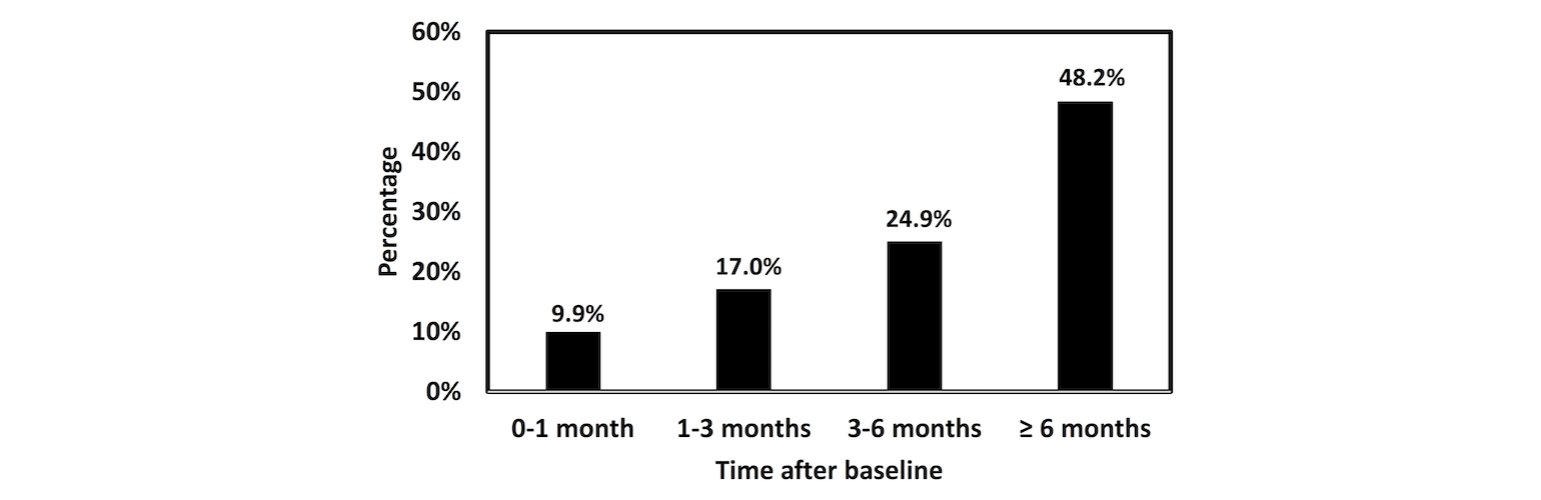
Distribution of high-risk patients in the validation cohort by time intervals between the model identification and coded chronic kidney disease diagnosis by International Classification of Disease, Ninth Revision, Clinical Modification.

Among high-risk patients in the validation cohort, 4177/52,594 received a new CKD diagnosis within the next 1 year. Figure 7 shows the distribution of the time intervals from the time point when a patient was identified as high risk by the model and the time point when the patient was assigned an ICD-9-CM CKD diagnosis code. Nearly half (48.24%) of the CKD cases were marked as high risk 6 months or more prior to assignment of a diagnosis code (ie, confirmatory diagnosis was made by physician).

## Discussion

### Principal Findings

We have derived and validated a risk model predictive of incident CKD diagnosis within the next 1 year across 1.3 million patients in the state of Maine. Through machine learning from preceding 1-year clinical profiles that were recorded in EMR databases, patients were classified into 3 risk categories (low, intermediate, and high risk), indicating the stratified probabilities of receiving CKD-related ICD-9-CM codes in the next 1 year. The model had similar performance in the derivation phase (*c*-statistic of 0.916) and validation phase (*c*-statistic of 0.871). Compared with other simplified score metrics [19,21,33], the model uses more predictors, giving a better result in classification (Multimedia Appendix 9). Performance of the model in subcohorts, especially those considered low risk by traditional risk factors (ie, age <65 years, no history of chronic disease) was fairly good (*c*-statistics 0.734 and 0.804, respectively), showing predictive power in patients with low awareness of CKD that traditional models tend to ignore. Model outcomes (Table 2) and survival analysis (Figure 6) both showed the model to provide reasonable risk stratification.

We applied a 2-step feature reduction process; 736 features survived after the first step (filtered by *P*<.05 plus literature review), and 146 features survived after the second step (filtered by non-zero weight in algorithm). Features having smaller *P* value in the chi-squared screening might not have larger weight in the algorithm due to the different mechanisms of establishing the relationship between the outcome and the features in the 2 steps of feature reduction. With this consideration, we set up *P*<.05 as a threshold to enable more features that might contribute to the modeling to go into the next step.

Results of misclassification analysis (Figure 5) show that 97.98% of false positives were patients who were ≥65 years of age or had a history of diabetes or hypertension. These patients, although they did not receive a CKD diagnosis within the next 1 year, were still considered at higher risk for developing CKD or other adverse outcomes than the general population. Monitoring these patients would help identify signs of CKD at an early stage and may benefit their long-term outcomes. Among false negatives, 99.61% were patients who lacked one or more major risk factor (eg, patients <65 years old or without a history of diabetes, hypertension, or kidney disorders), causing the model to identify them as low or intermediate risk.

Our model identified patients in other or unknown race categories as less likely to get CKD. The percentages of patients in the other race category were 16.07% (203,546/1,266,983) in the low-risk category and 0.16% (86/52,594) in the high-risk category in the validation cohort. Most patients (90.13%, 183,723/203,847) in the other or unknown race category actually had an unknown race marked in the dataset. It perhaps indicated a data quality issue that the race information was probably missing. Compared to the total studied population, patients in the unknown race category had a much lower rates of history of diabetes (0.16% vs 4.24%) and hypertension (0.38% vs 9.32%), and fewer outpatient visits (5.59% vs 56.04%). Lack of risk factors of CKD made the majority of these patients stratified to the low-risk group. However, such results didn’t mean these patients were healthier than the average level of the total population. As described in the Multimedia Appendix 2, for a patient who didn’t have any EMR, it is hard to tell whether this patient was healthy thus had never used care service or this patient did use care service but the records were missing. Such limitation was caused by the EMR storage format.

A main challenge of this study was that actual values of estimated glomerular filtration rate (eGFR) and albumin-to-creatinine ratio (ACR), the 2 parameters used to determine CKD stage [34,35], were not available in our data source. The total counts of abnormal creatinine blood test results and ACR over the preceding 1-year period were used instead. Moreover, as it was a study on the general population, most of the participants did not have abnormal test results related to eGFR or ACR. Therefore, unlike other studies in which eGFR and ACR played critical roles in CKD prediction, these parameters were not selected as top features by the model proposed in this study. The model, however, had performance comparable to studies using exact values of eGFR and ACR as predictors [21,22], indicating that CKD incidence can be predicted without knowledge of eGFR or ACR. These results support the potential value of EMR- or claims-based retrospective studies in which actual laboratory test results tend to be missing due to data quality issues or data sharing policies. An analysis of Medicare patients showed that even among patients older than 65 years, a group at high risk for CKD, less than 80% of patients had claims indicating serum creatinine testing and less than 20% had urine albumin testing [3]. Development and validation of a CKD risk model within a general population in which eGFR and ACR are frequently absent is extremely useful for its applicability in clinical practice as a routinely used assistant tool. It makes our model an economically feasible method for general population screening because it eliminates the time and costs of collecting eGFR and ACR during traditional screening tests of CKD [36-38]. A prescreening on general population using the proposed model followed by tests of urine albumin and serum creatinine on high-risk patients forms a cost-effective approach to identify risks of CKD.

Another challenge was that this study targeted prediction of CKD incidence within the next 1 year, which is a short time horizon compared with other studies of CKD prediction in which the follow-up periods were several years [21-23]. Such a short time frame resulted in a low incidence (0.57% in the derivation cohort and 0.58% in validation cohort), which increased the difficulty of prediction. The complex model with multiple predictors allowed identification of a group of patients with a high 1-year incidence of CKD (derivation phase 11.82%, 20.7 times higher than the baseline; validation phase 7.94%, 13.7 times higher than the baseline). These patients were labeled as high risk and are good targets for administration and intervention plans. Traditional risk factors (age, history of diabetes and hypertension) identified a group of patients with a 1-year incidence of 1.95% in the derivation phase and 1.97% in the validation phase, only about 3 times higher than the baseline.

### Interpretation of Predictors

The feature selection process that combined both data-driven methodology and domain knowledge resulted in a list of predictors composing the predictive algorithm (Multimedia Appendix 5). Traditional risk factors of CKD remained highly important. Age and the use of furosemide were 2 predictors of top importance. This observation makes sense, as age is considered a common risk factor of CKD, while furosemide is a medication used in patients with congestive heart disease, kidney disorders, and high blood pressure, all of which are correlated with CKD. The link between cardiovascular diseases and CKD has been reported in many studies, and the role of cardiovascular diseases in the development and progression of CKD was found [39,40]. CKD was found in over half of patients with heart failure [41]. CKD and cardiovascular diseases share common risk factors, and a bidirectional pathway was noticed between the progression of cardiovascular disease and CKD [39]. Medical history of furosemide, which is commonly used to treat congestive heart failure, therefore may indicate a risk of CKD initiation. What’s more, furosemide is a commonly used preventive and therapeutic drug for acute kidney injury (AKI) [42]. The benefits of furosemide in reducing hypertension and improving eGFR show its potential role in reducing the risk of AKI. Compared with other diuretics for kidney diseases such as bumetanide, hydrochlorothiazide, and spironolactone that were predictors of our model, furosemide is more powerful and less expensive. The biological link between AKI and CKD has been established, and AKI is considered as an independent risk factor of CKD.

In addition, the model identified a group of previously prescribed medications as predictors, primarily drugs for diabetes (insulin glargine, insulin isophane, glipizide, insulin fetemir, etc.), blood pressure control (hydralazine, amlodipine besylate, metoprolol tartrate, etc.), heart diseases (isosorbide mononitrate, valsartan, amiodarone, etc.), and kidney disorders (allopurinol). Such medication histories indicate patients either at risk for or living with diseases that might lead to CKD. Prescriptions for medications used for inflammatory processes (prednisone and colchicine), bone disease (febuxostat), anemia (folic acid), and hypokalemia (potassium chloride) were also identified as predictors, illustrating their contribution to the disease network. Abnormal results of metabolic panel, glucose test, coagulation test, and therapeutic drug monitoring were predictors in the laboratory test category, which indicates disease states such as diabetes. History of hypertension, renal disorders, heart diseases, anemia, and diabetes were top important diagnostic features that were highly correlated to CKD.

In addition to the clinical features, variables indicative of high resource consumption (eg, health care costs, total counts of medications, laboratory and radiology tests, outpatient visits, and inpatient length of stay) were also considered risk factors by the model. This pattern identifies heavy users of health care services (eg, older patients or patients with multiple chronic morbidities) to have a higher probability of developing CKD, which makes sense as CKD has been considered as a complication of complex chronic diseases that are associated with large health resource expenditures [3].

In all, senior patients and heavy users of care resources with chronic conditions like diabetes and hypertension that are highly correlated to CKD tend to be classified as high risk for incident CKD by the model.

### Beyond Risk Estimation

Several previous studies have reported the development and validation of CKD risk scores. The predictors, modeling process, validation, and accuracy of the scores were well presented, but little effort was made to translate the risk scores to patient care action plans. Those studies addressed whether the risk of CKD onset or progression can be predicted but did not address what actions should be taken for high-risk patients [43]. The widespread application of EMR in the state of Maine has enabled us to develop risk scores for the Maine residents [27,28,44-46] in terms of future resource utilization and clinical conditions. The meaningful use of EMR data, however, is not only to forecast the health status in the future but also to guide the health care providers to make decisions in the present. There are already established guidelines in CKD preventive care to address both nonmodifiable and modifiable risk factors. For example, CKD screening is recommended on a regular basis for patients with nonmodifiable risk factors (eg, older patients) to identify CKD at an early stage.

For patients with modifiable risk factors such as concurrent chronic conditions, life styles, and medications, there are quite a few targeted intervention options to reduce the risk. Nutritional treatments such as a low-protein diet together with sufficient and regular exercise should be initiated on patients with obesity, hypertension, or diabetes to prevent or slow CKD progression [47,48]. Medications that may reduce renal function or cause complications, such as angiotensin-converting enzyme and nonsteroidal anti-inflammatory drugs, should be prescribed with careful consideration and monitoring plans if necessary [49,50]. Advice to stop smoking and limit alcohol should be given to smokers and alcohol users to improve overall health and reduce the risk of CKD [51,52] for those individuals. The modifiable risk factors are even more important than nonmodifiable predictors as they offer an opportunity to both clinicians and patients to be proactive to the disease by implementing interventions before deterioration.

In all, a combination of a single scalar score and longitudinal clinical profile including chronic disease history, current problem list, and therapies and medications will help clinicians develop a personalized action plan with modifiable risk factors for each high-risk individual. It is the subsequent actions rather than an isolated risk score that help improve health status, outcomes, and resource utilization [53]. The ultimate goal of this study is to confirm, modify, or disapprove care plans based on the risk prediction outcomes, leading to improved quality of care. Obtaining a risk score is not the end of the study but the first step of translating predictive analytics into prescriptive solutions, a proactive approach to prevent or delay deterioration in health.

### Implications for Treatment and Prognosis

A chart showing time intervals between identification of high-risk patients and receiving a CKD diagnosis code in Figure 7 reveals clinical implications for treatment and prognosis of CKD. Certain interventions at an early stage can reduce the risk of developing CKD or progression to end-stage disease. For example, clinical trials showed that patients receiving blood pressure control treatment had significantly reduced proteinuria within the first 4 months compared with those had no blood pressure control, suggesting a reduced risk of CKD development and progression [54,55]. A meta-analysis reported that lifestyle modifications for 3 months decreased the risk for diabetes from the end of intervention up to 10 years later [56], which in turn correlated to attenuated risk of developing CKD, as diabetes has been recognized as an important predisposing factor for CKD.

In our validation cohort, the model identified 72.90% (3045/4177) of high-risk patients at least 3 months before the confirmatory diagnosis was made by physicians. Of those patients, 41.02% (1249/3045) had diabetes or an abnormal glucose test result at the time they were identified by the model to be at high risk for CKD. Implementation of lifestyle modifications at that time has the potential to mitigate adverse outcomes in those patients. Moreover, 64.59% (2698/4177) of high-risk patients were identified by the model at least 4 months prior to confirmatory diagnosis, and 9.82% (265/2698) of those patients were not taking any blood pressure medication and did not have a diagnosis of hypertension. Blood pressure monitoring and necessary control in these patients can help to reduce the risk of CKD. Such explorations highlight potential meaningful use of the model in clinical practice, in that it can help to initiate decision making and timely intervention.

The predictive model and risk scores can benefit health care organizations at multiple levels. For health care managers who take charge of the population management at the whole department or hospital, the population stratification by risk scores will help with budget planning, as high-risk patients tend to require more resources. For physicians, the model can be used as an assistant tool for decision making. High-risk patients without eGFR or ACR parameters available can be referred to the CKD screening test to decide whether or not the patients have CKD already. The risk stratification will also give physicians ideas of treating patients at high risk of CKD for other concurrent clinical problems, especially in the situation where the current medical or surgical treatments can help with the existing problems but accelerate CKD progression in patients. Clinicians can also drill down to see what information is driving the risk scores, which provides the clinical background they need to trust and act on the risk scores.

### Study Limitations

There are several limitations of this study. First, uncoded CKD cases could be outliers of the model and affect accuracy. Patients with undiagnosed CKD might not be excluded from the study cohort. Computed false positives from undiagnosed CKD patients missing diagnosis codes are actually true positives, especially for those who were over age 65 years. eGFRs of older patients tend to be lower and thus may further complicate the diagnosis. Patients who were waiting a random urine test for confirmative diagnosis but didn’t have an ICD-9-CM code assigned during the study period could confound the model as well. Maine HIE went live in 2009, so patients with CKD diagnosed before 2009 might not be documented in the EMR database. These patients could be treated as false positives during the performance evaluation. Second, there might be a delay of the assignment of an ICD-9-CM code that was longer than the transition of kidney function from a normal state to a disease state. It might explain why the incidence rate (0.568%-0.580%) in our study cohort was higher than reported by other studies [57,58], as some of the patients who received an ICD-9-CM code might already have undiagnosed stage 1 to 2 CKD. Assignment of an ICD-9-CM code doesn’t always mean a new case showing up. Another possible reason for the high incidence was that the study cohort had a slight age bias (20.96% in the validation cohort vs 15.64% in the overall population in Maine for percentage of patients at 65 years and over), and age is an independent risk factor of CKD. Third, unlike other CKD risk models, our model does not include exact values of eGFR or ACR as predictors due to lack of such data, and it is possible that including eGFR could further improve the model performance. Fourth, all laboratory test variables were labeled as either normal or abnormal in the data source. A detailed classification of laboratory test results would help to construct a deeper understanding of clinical conditions of patients and enhance model performance. Fifth, due to the nature of EMR storage format, we cannot differentiate the situation where a particular medical record was missing, although it would happen at a very low probability. Sixth, the cutoff point (score ≥ 0.05) for high-risk categories was selected to optimize the PPV with a fair value of sensitivity. In the production dashboard we deployed at the Maine HIE, there is an option to allow each provider user to set up its own cutoffs on our real-time population health care surveillance platform. Seventh, all the study participants were from the state of Maine, and recalibration as well as other necessary adjustments would be needed before leveraging the model to health care management for populations in other states. Geographical, environmental, and racial disparities may contribute to population characteristics, and additional risk factors should be considered if necessary.

### Conclusions

A risk model that estimated the probability of receiving CKD diagnosis within the next 1 year was developed and validated in this study. Through the statistical learning of the EMRs of over 1.3 million patients in the state of Maine, the model was able to assign each individual a risk score based on the preceding 1-year clinical history. The whole population was stratified into 3 risk categories according to the score, where the high-risk category had a CKD incidence 13.7 times higher than the baseline. A *c*-statistic of 0.871 was achieved in the validation phase. Identification of patients at high risk of receiving CKD diagnosis will help to promote care plans of monitoring and intervention, which will ultimately benefit the outcomes of patients.

## Appendix

Multimedia Appendix 1 Transparent Reporting of a Multivariable Prediction Model for Individual Prognosis or Diagnosis (TRIPOD) checklist for reporting derivation and validation predictive model.

Multimedia Appendix 2 Missing data handling.

Multimedia Appendix 3 Construction of derivation and validation cohorts.

Multimedia Appendix 4 List of 736 features considered for model derivation.

Multimedia Appendix 5 List of 146 features (final predictors) and their weights in the model.

Multimedia Appendix 6 Top 50 features (ie, predictors) in the final model: weight, log odds ratio, and 0.95 confidence interval.

Multimedia Appendix 7 A 2-by-2 contingency table for the derivation cohort and the validation cohort with a cutoff score of 0.05.

Multimedia Appendix 8 Relationships between sensitivities, specificities, and positive predictive values of the model on the validation cohort.

Multimedia Appendix 9 Comparison of c-statistics using other clinical prediction scores.

## Abbreviations

ACR: albumin-to-creatinine ratio
AKI: acute kidney injury
CKD: chronic kidney disease
eGFR: estimated glomerular filtration rate
EMR: electronic medical record
HIE: Health Information Exchange
ICD-9-CM: International Classification of Diseases, Ninth Revision, Clinical Modification
NHANES: National Health and Nutrition Examination Survey
PPV: positive predictive value
ROC: receiver operating characteristic
TRIPOD: Transparent Reporting of a Multivariable Prediction Model for Individual Prognosis or Diagnosis
