# Supplementary material for: Estimating One-Year Risk of Incident Chronic Kidney Disease: Retrospective Development and Validation Study Using Electronic Medical Record Data From the State of Maine
Source: JMIR Med Inform. 2017 Jul 26;5(3):e21. doi: 10.2196/medinform.7954 (PMC5550735; doi:10.2196/medinform.7954)
Supplement: Multimedia Appendix 1 [file medinform_v5i3e21_app1.pdf]

Multimedia appendix 1. TRIPOD checklist for reporting derivation and validation predictive model

Section/topic	Item	Development or validation?	Checklist item	Page
**Title and abstract**				
Title	1	D;V	Identify the study as developing and/or validating a multivariable prediction model, the target population, and the outcome to be predicted	Title
Abstract	2	D;V	Provide a summary of objectives, study design, setting, participants, sample size, predictors, outcome, statistical analysis, results, and conclusions	Abstract
**Introduction**				
Background and objectives	3a	D;V	Explain the medical context (including whether diagnostic or prognostic) and rationale for developing or validating the multivariable prediction model, including references to existing models	Introduction
	3b	D;V	Specify the objectives, including whether the study describes the development or validation of the model, or both	Introduction
**Methods**				
Source of data	4a	D;V	Describe the study design or source of data (for example, randomised trial, cohort, or registry data), separately for the development and validation data sets, if applicable	Methods-Studied population and source of data
	4b	D;V	Specify the key study dates, including start of accrual; end of accrual; and, if applicable, end of follow-up	Methods-Studied population and source of data
Participants	5a	D;V	Specify key elements of the study setting (for example, primary care, secondary care, general population) including number and location of centres	Methods-Studied population and source of data

	5b	D;V	Describe eligibility criteria for participants	Methods-Studied population and source of data
	5c	D;V	Give details of treatments received, if relevant	N/A
Outcome	6a	D;V	Clearly define the outcome that is predicted by the prediction model, including how and when assessed	Methods-Outcome definition
	6b	D;V	Report any actions to blind assessment of the outcome to be predicted	N/A
Predictors	7a	D;V	Clearly define all predictors used in developing the multivariable prediction model, including how and when they were measured	Methods-Studied population and source of data; Methods-Feature reduction
	7b	D;V	Report any actions to blind assessment of predictors for the outcome and other predictors	N/A
Sample size	8	D;V	Explain how the study size was arrived at.	Methods-Studied population and source of data
Missing data	9	D;V	Describe how missing data were handled (for example, complete-case analysis, single imputation, multiple imputation) with details of any imputation method	Multimedia Appendix 2
Statistical analysis methods	10a	D	Describe how predictors were handled in the analyses	Methods-Feature reduction
	10b	D	Specify type of model, all model-building procedures (including any predictor selection), and method for internal validation	Methods-Derivation phase
	10c	V	For validation, describe how the predictions were calculated	Methods-Validation phase
	10d	D;V	Specify all measures used to assess model performance and, if relevant, to compare multiple models	Methods-Derivation phase; Methods-Validation phase
	10e	V	Describe any model updating (for example, recalibration) arising from the validation, if	N/A

			done	
Risk groups	11	D;V	Provide details on how risk groups were created, if done	Methods-Validation phase
Development *v* validation	12	V	For validation, identify any differences from the development data in setting, eligibility criteria, outcome, and predictors	Methods-Validation phase
**Results**				
Participants	13a	D;V	Describe the flow of participants through the study, including the number of participants with and without the outcome and, if applicable, a summary of the follow-up time. A diagram may be helpful	Results-Study cohort
	13b	D;V	Describe the characteristics of the participants (basic demographics, clinical features, available predictors), including the number of participants with missing data for predictors and outcome	Results-Baseline characteristics; Table 1
	13c	V	For validation, show a comparison with the development data of the distribution of important variables (demographics, predictors and outcome).	Results-Baseline characteristics; Table 1
Model development	14a	D	Specify the number of participants and outcome events in each analysis	Results-Derivation phase
	14b	D	If done, report the unadjusted association between each candidate predictor and outcome	N/A
Model specification	15a	D	Present the full prediction model to allow predictions for individuals (that is, all regression coefficients, and model intercept or baseline survival at a given time point)	Multimedia Appendix 5
	15b	D	Explain how to use the prediction model	Results-Derivation phase
Model	16	D;V	Report performance	Table 2

performance			measures (with CIs) for the prediction model	
Model updating	17	V	If done, report the results from any model updating (that is, model specification, model performance)	Results-Validation phase
**Discussion**				
Limitations	18	D;V	Discuss any limitations of the study (such as nonrepresentative sample, few events per predictor, missing data)	Discussion-Study limitations
Interpretation	19a	V	For validation, discuss the results with reference to performance in the development data, and any other validation data	Discussion-Summary of main findings
	19b	D;V	Give an overall interpretation of the results, considering objectives, limitations, results from similar studies, and other relevant evidence	Discussion-Summary of main findings; Discussion-Studied limitation
Implications	20	D;V	Discuss the potential clinical use of the model and implications for future research	Discussion-Interpretation of predictors; Discussion-Implications for treatment and prognosis
