# Supplementary material for: Estimating One-Year Risk of Incident Chronic Kidney Disease: Retrospective Development and Validation Study Using Electronic Medical Record Data From the State of Maine
Source: JMIR Med Inform. 2017 Jul 26;5(3):e21. doi: 10.2196/medinform.7954 (PMC5550735; doi:10.2196/medinform.7954)
Supplement: Multimedia Appendix 2 [file medinform_v5i3e21_app2.pdf]

Multimedia appendix 2. Missing data handling

Inevitably some data were missing for some records in our data warehouse. It was a data integrity problem that existed before we obtained data from HIE. There were around 3.4% of encounters with missing geographic information, 0.68% with missing gender, and 0.20% with missing admission or discharge date in our database. Encounter records with missing demographics were removed from our study cohort. There were possibly some longitudinal data of clinical histories missing as well. However, it was hard to tell from the database whether such information was actually missing or not. For example, having an inpatient admission count of 0 in prior one year may indicate that the patient didn’t visit a hospital during last year, or the admission information for this patient was not transferred to the HIE (though it would happen at a very low probability). It is a study limitation.
