# Supplementary figures and images for: Estimating One-Year Risk of Incident Chronic Kidney Disease: Retrospective Development and Validation Study Using Electronic Medical Record Data From the State of Maine
Source: JMIR Med Inform. 2017 Jul 26;5(3):e21. doi: 10.2196/medinform.7954 (PMC5550735; doi:10.2196/medinform.7954)

### Multimedia appendix 3. Construction of derivation and validation cohorts

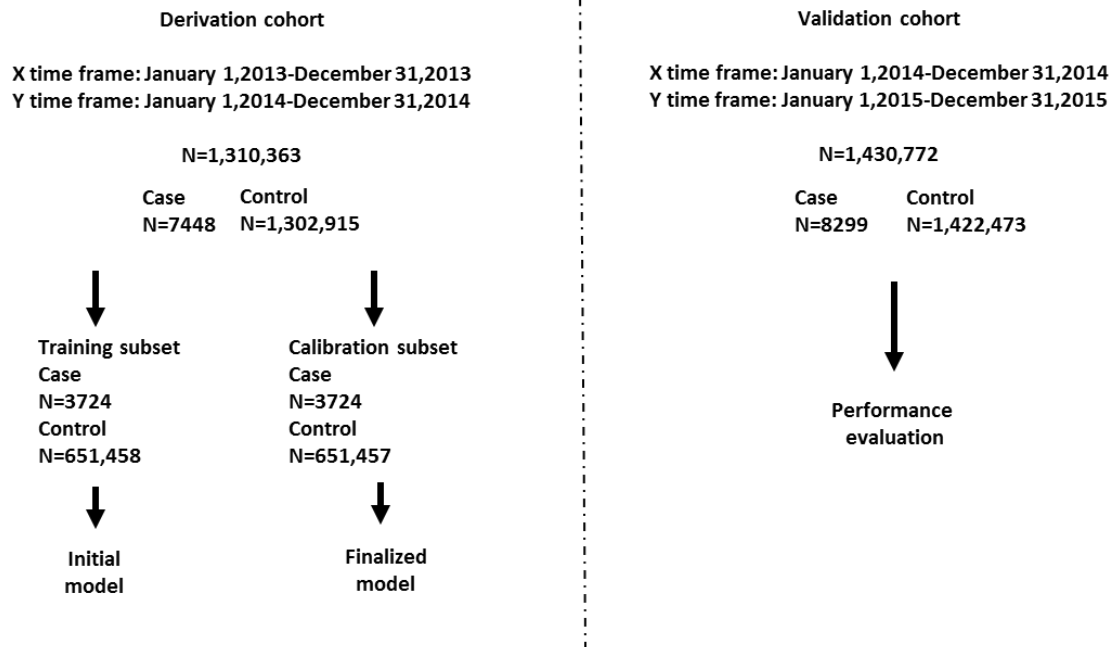

Supplement: Multimedia Appendix 3 [file medinform_v5i3e21_app3.pdf]
