# Supplementary material for: Estimating One-Year Risk of Incident Chronic Kidney Disease: Retrospective Development and Validation Study Using Electronic Medical Record Data From the State of Maine
Source: JMIR Med Inform. 2017 Jul 26;5(3):e21. doi: 10.2196/medinform.7954 (PMC5550735; doi:10.2196/medinform.7954)
Supplement: Multimedia Appendix 4 [file medinform_v5i3e21_app4.pdf]

Multimedia appendix 4. A list of 736 features considered for model derivation

No.	Feature
1	1 ml insulin syringes with needle
2	Abdomen and pelvis CT without co contrast
3	Abdomen narrative x-ray
4	Abdomen us limited
5	Abdomen x-ray ap (supine and upright)
6	Abdominal and Pelvic CT with contrast
7	Acetic acid
8	Activated partial thromboplastin time (APTT) in platelet poor plasma by coagulation assay_abnormal
9	Acute tubular necrosis
10	Acyclovir
11	Age
12	Albuterol sulfate
13	Alcohol antiseptic pads
14	Aliskiren hemifumarate
15	Allopurinol
16	Amiodarone
17	Amitriptyline hcl
18	Amlodipine besylate
19	Amoxicillin
20	Anemia
21	Arteriovenous shunt dialysis report
22	Aspirin
23	Aspirin/dipyridamole
24	Atacand
25	Atenolol
26	Atherosclerotic heart disease (ASHD)
27	atorvastatin (Lipitor)
28	Atorvastatin calcium
29	Avapro
30	Azithromycin
31	Basic metabolic 2000 panel - serum or plasma_abnormal
32	Basic metabolic panel - Blood_abnormal
33	Basic metabolic panel_abnormal
34	Basic metabolic panel-ew only_abnormal
35	Benazepril (Lotensin)
36	Benicar
37	Bimatoprost
38	Blood Gas and Metabolites_abnormal
39	Blood gas, arterial_abnormal
40	Blood Gas, Venous_abnormal
41	Blood sugar diagnostic
42	Blood Urea Nitrogen-higher than normal

43 Blood Urea Nitrogen-lower than normal
44 Blood Urea Nitrogen-normal
45 Blue Cross beneficiary
46 Boceprevir
47 Bosentan
48 Brachiocephalic artery fluoroscopic angiogram angioplasty with contrast ia
49 Breast - bilateral mammogram
50 Breast ffd mammogram screening
51 Brimonidine tartrate
52 Budesonide/formoterol fumarate
53 Bumetanide
54 Bupropion hcl
55 Calcitriol
56 Calcium acetate
57 Calcium carbonate (Tums)
58 Calcium supplement
59 Cancer
60 Candesartan cilexetil
61 Candesartan/hydrochlorothiazid
62 Captopril (Capoten)
63 Cardiac, other
64 Cardiovascular disease, unspecified
65 Carisoprodol
66 Carvedilol
67 CBC with differential panel, method unspecified - blood_abnormal
68 CBC with ordered manual differential panel - blood_abnormal
69 CBC with reflex manual differential panel - blood_abnormal
70 CBC with auto differential panel in blood_abnormal
71 CBC with Differential_abnormal
72 CBC with Ordered Manual Differential panel in Blood_abnormal
73 CBC/Manual Differential, Blood_abnormal
74 Cephalexin
75 Cerebrovascular accident (CVA) / transient ischemic attack (TIA)
76 Chem 6_abnormal
77 Chem 8_abnormal
78 Chemistry profile_abnormal
79 Chest pain
80 Chest x-ray 2 views
81 Chest x-ray pa and lateral
82 Chest x-ray portable
83 chlorothiazide [(Diuril) (oral or sodium injection)]
84 chlorthalidone (Hygroton)
85 Chronic condition_Acquired foot deformities
86 Chronic condition_Acute and chronic tonsillitis
87 Chronic condition_Acute and unspecified renal failure
88 Chronic condition_Acute cerebrovascular disease

89	Chronic condition_Acute myocardial infarction
90	Chronic condition_Alcohol-related disorders
91	Chronic condition_Allergic reactions
92	Chronic condition_Anxiety disorders
93	Chronic condition_Aortic and peripheral arterial embolism or thrombosis
94	Chronic condition_Aortic; peripheral; and visceral artery aneurysms
95	Chronic condition_Asthma
96	Chronic condition_Biliary tract disease
97	Chronic condition_Blindness and vision defects
98	Chronic condition_Cancer of bladder
99	Chronic condition_Cancer of bone and connective tissue
100	Chronic condition_Cancer of brain and nervous system
101	Chronic condition_Cancer of breast
102	Chronic condition_Cancer of bronchus; lung
103	Chronic condition_Cancer of cervix
104	Chronic condition_Cancer of colon
105	Chronic condition_Cancer of esophagus
106	Chronic condition_Cancer of head and neck
107	Chronic condition_Cancer of kidney and renal pelvis
108	Chronic condition_Cancer of liver and intrahepatic bile duct
109	Chronic condition_Cancer of other female genital organs
110	Chronic condition_Cancer of other GI organs; peritoneum
111	Chronic condition_Cancer of other male genital organs
112	Chronic condition_Cancer of other urinary organs
113	Chronic condition_Cancer of ovary
114	Chronic condition_Cancer of pancreas
115	Chronic condition_Cancer of prostate
116	Chronic condition_Cancer of rectum and anus
117	Chronic condition_Cancer of stomach
118	Chronic condition_Cancer of testis
119	Chronic condition_Cancer of thyroid
120	Chronic condition_Cancer of uterus
121	Chronic condition_Cancer; other and unspecified primary
122	Chronic condition_Cancer; other respiratory and intrathoracic
123	Chronic condition_Cardiac and circulatory congenital anomalies
124	Chronic condition_Cardiac arrest and ventricular fibrillation
125	Chronic condition_Cardiac dysrhythmias
126	Chronic condition_Cataract
127	Chronic condition_Chronic kidney disease
128	Chronic condition_Chronic obstructive pulmonary disease and bronchiectasis
129	Chronic condition_Chronic ulcer of skin
130	Chronic condition_Coagulation and hemorrhagic disorders
131	Chronic condition_Coma; stupor; and brain damage
132	Chronic condition_Complication of device; implant or graft
133	Chronic condition_Complications of surgical procedures or medical care
134	Chronic condition_Conditions associated with dizziness or vertigo

135 Chronic condition_Conduction disorders
136 Chronic condition_Congestive heart failure; nonhypertensive
137 Chronic condition_Coronary atherosclerosis and other heart disease
138 Chronic condition_Cystic fibrosis
139 Chronic condition_Deficiency and other anemia
140 Chronic condition_Delirium, dementia, and amnestic and other cognitive disorders
141 Chronic condition_Diabetes mellitus with complications
142 Chronic condition_Diabetes mellitus without complication
143 Chronic condition_Diabetes or abnormal glucose tolerance complicating pregnancy; childbirth; or the puerperium
144 Chronic condition_Digestive congenital anomalies
145 Chronic condition_Diseases of white blood cells
146 Chronic condition_Disorders of lipid metabolism
147 Chronic condition_Disorders of teeth and jaw
148 Chronic condition_Diverticulosis and diverticulitis
149 Chronic condition_Encephalitis (except that caused by tuberculosis or sexually transmitted disease)
150 Chronic condition_Endometriosis
151 Chronic condition_Epilepsy; convulsions
152 Chronic condition_Esophageal disorders
153 Chronic condition_Essential hypertension
154 Chronic condition_Female infertility
155 Chronic condition_Gangrene
156 Chronic condition_Gastritis and duodenitis
157 Chronic condition_Gastroduodenal ulcer (except hemorrhage)
158 Chronic condition_Gastrointestinal hemorrhage
159 Chronic condition_Genitourinary congenital anomalies
160 Chronic condition_Genitourinary symptoms and ill-defined conditions
161 Chronic condition_Glaucoma
162 Chronic condition_Gout and other crystal arthropathies
163 Chronic condition_Headache; including migraine
164 Chronic condition_Heart valve disorders
165 Chronic condition_Hepatitis
166 Chronic condition_HIV infection
167 Chronic condition_Hodgkin`s disease
168 Chronic condition_Hyperplasia of prostate
169 Chronic condition_Hypertension complicating pregnancy; childbirth and the puerperium
170 Chronic condition_Hypertension with complications and secondary hypertension
171 Chronic condition_Immunity disorders
172 Chronic condition_Infective arthritis and osteomyelitis (except that caused by tuberculosis or sexually transmitted disease)
173 Chronic condition_Inflammation; infection of eye (except that caused by tuberculosis or sexually transmitteddisease)

174	Chronic condition_Inflammatory conditions of male genital organs
175	Chronic condition_Inflammatory diseases of female pelvic organs
176	Chronic condition_Joint disorders and dislocations; trauma-related
177	Chronic condition_Late effects of cerebrovascular disease
178	Chronic condition_Leukemias
179	Chronic condition_Lung disease due to external agents
180	Chronic condition_Lymphadenitis
181	Chronic condition_Maintenance chemotherapy; radiotherapy
182	Chronic condition_Malaise and fatigue
183	Chronic condition_Malignant neoplasm without specification of site
184	Chronic condition_Melanomas of skin
185	Chronic condition_Menopausal disorders
186	Chronic condition_Menstrual disorders
187	Chronic condition_Miscellaneous disorders
188	Chronic condition_Mood disorders
189	Chronic condition_Multiple myeloma
190	Chronic condition_Multiple sclerosis
191	Chronic condition_Neoplasms of unspecified nature or uncertain behavior
192	Chronic condition_Nephritis; nephrosis; renal sclerosis
193	Chronic condition_Nervous system congenital anomalies
194	Chronic condition_Non-Hodgkin`s lymphoma
195	Chronic condition_Noninfectious gastroenteritis
196	Chronic condition_Nonmalignant breast conditions
197	Chronic condition_Nutritional deficiencies
198	Chronic condition_Occlusion or stenosis of precerebral arteries
199	Chronic condition_Open wounds of extremities
200	Chronic condition_Osteoarthritis
201	Chronic condition_Osteoporosis
202	Chronic condition_Other acquired deformities
203	Chronic condition_Other aftercare
204	Chronic condition_Other and ill-defined cerebrovascular disease
205	Chronic condition_Other and ill-defined heart disease
206	Chronic condition_Other and unspecified benign neoplasm
207	Chronic condition_Other bone disease and musculoskeletal deformities
208	Chronic condition_Other circulatory disease
209	Chronic condition_Other CNS infection and poliomyelitis
210	Chronic condition_Other complications of pregnancy
211	Chronic condition_Other congenital anomalies
212	Chronic condition_Other connective tissue disease
213	Chronic condition_Other diseases of bladder and urethra
214	Chronic condition_Other diseases of kidney and ureters
215	Chronic condition_Other diseases of veins and lymphatics
216	Chronic condition_Other disorders of stomach and duodenum
217	Chronic condition_Other ear and sense organ disorders
218	Chronic condition_Other endocrine disorders
219	Chronic condition_Other eye disorders

220 Chronic condition_Other female genital disorders
221 Chronic condition_Other gastrointestinal disorders
222 Chronic condition_Other hematologic conditions
223 Chronic condition_Other hereditary and degenerative nervous system conditions
224 Chronic condition_Other infections; including parasitic
225 Chronic condition_Other inflammatory condition of skin
226 Chronic condition_Other liver diseases
227 Chronic condition_Other lower respiratory disease
228 Chronic condition_Other male genital disorders
229 Chronic condition_Other nervous system disorders
230 Chronic condition_Other non-epithelial cancer of skin
231 Chronic condition_Other non-traumatic joint disorders
232 Chronic condition_Other nutritional; endocrine; and metabolic disorders
233 Chronic condition_Other perinatal conditions
234 Chronic condition_Other skin disorders
235 Chronic condition_Other upper respiratory disease
236 Chronic condition_Other upper respiratory infections
237 Chronic condition_Otitis media and related conditions
238 Chronic condition_Pancreatic disorders (not diabetes)
239 Chronic condition_Paralysis
240 Chronic condition_Parkinson`s disease
241 Chronic condition_Peri-; endo-; and myocarditis; cardiomyopathy (except that caused by tuberculosis or sexually transmitted disease)
242 Chronic condition_Peripheral and visceral atherosclerosis
243 Chronic condition_Phlebitis; thrombophlebitis and thromboembolism
244 Chronic condition_Pleurisy; pneumothorax; pulmonary collapse
245 Chronic condition_Pneumonia (except that caused by tuberculosis or sexually transmitted disease)
246 Chronic condition_Poisoning by nonmedicinal substances
247 Chronic condition_Prolapse of female genital organs
248 Chronic condition_Pulmonary heart disease
249 Chronic condition_Regional enteritis and ulcerative colitis
250 Chronic condition_Rehabilitation care; fitting of prostheses; and adjustment of devices
251 Chronic condition_Residual codes; unclassified
252 Chronic condition_Respiratory failure; insufficiency; arrest (adult)
253 Chronic condition_Retinal detachments; defects; vascular occlusion; and retinopathy
254 Chronic condition_Rheumatoid arthritis and related disease
255 Chronic condition_Schizophrenia and other psychotic disorders
256 Chronic condition_Screening and history of mental health and substance abuse codes
257 Chronic condition_Secondary malignancies
258 Chronic condition_Sexually transmitted infections (not HIV or hepatitis)
259 Chronic condition_Sickle cell anemia

260 Chronic condition_Spinal cord injury
261 Chronic condition_Spondylosis; intervertebral disc disorders; other back problems
262 Chronic condition_Substance-related disorders
263 Chronic condition_Systemic lupus erythematosus and connective tissue disorders
264 Chronic condition_Thyroid disorders
265 Chronic condition_Transient cerebral ischemia
266 Chronic condition_Tuberculosis
267 Chronic condition_Urinary tract infections
268 Chronic condition_Viral infection
269 Chronic obstructive pulmonary disorder (COPD)
270 Cilostazol
271 Cinacalcet (Sensipar)
272 Citalopram hydrobromide
273 Clonazepam
274 Clopidogrel bisulfate
275 Clotrimazole and betamethasone dipropionate
276 Codeine/promethazine hcl
277 Colchicine
278 Collagenase clostridium hist.
279 Commercial health insurance beneficiary
280 Complete blood count (hemogram) panel - blood by automated count_abnormal
281 Complete blood count with differential_abnormal
282 Complete blood count_abnormal
283 Comprehensive metabolic 2000 panel - serum or plasma_abnormal
284 Comprehensive metabolic panel_abnormal
285 Congestive heart failure (CHF)
286 Cozaar
287 Creatinine panel_abnormal
288 Cyanocobalamin/fa/pyridoxine
289 Cyclobenzaprine hcl
290 Cyclosporine, modified
291 Dabigatran etexilate mesylate
292 Darbepoetin (Aranesp)
293 Decreased urine output
294 Dexlansoprazole
295 Diabetes mellitus (DM)
296 Differential automated_abnormal
297 Differential_abnormal
298 Digoxin
299 Digoxin [Mass/volume] in Serum or Plasma_abnormal
300 Diltiazem hcl
301 Diovan
302 Dipyridamole

303 Disposable insulin needles
304 Doxazosin mesylate
305 Doxercalciferol (Hectorol)
306 Dutasteride
307 Dysrhythmia
308 edema
309 Electrophoresis, Random Urine_abnormal
310 Enalapril/Enalaprilat (Vasotec oral and injectable)
311 Enlarged prostate
312 Epoetin alfa
313 Epoetin alfa (Epogen)
314 Epoetin alfa (Procrit)
315 Eszopiclone
316 Exenatide microspheres
317 Ezetimibe
318 Family history of kidney disease
319 Famotidine
320 Febuxostat
321 Felodipine
322 Fenofibrate
323 Fenofibrate nanocrystallized
324 Fenofibric acid (choline)
325 Fentanyl
326 Ferric carboxymaltose (Injectafer)
327 Ferric gluconate (Ferrlecit)
328 Ferric gluconate (Nulecit)
329 Ferric pyrophosphate (Triferic)
330 Ferrous sulfate
331 Ferumoxytol (Feraheme)
332 Finasteride
333 Fluocinolone acetonide
334 Fluoxetine hcl
335 Fluticasone propionate
336 Fluticasone/salmeterol
337 fluvastatin (Lescol, Lescol XL)
338 Folic acid
339 Folic acid/vitamin b comp w-c
340 Fosfomycin tromethamine
341 Fosinopril sodium
342 Furosemide
343 Gabapentin
344 Gas panel - arterial blood_abnormal
345 Gastrointestinal bleeding disorders (GI)
346 Gemfibrozil
347 Gender (Male)
348 Gentamicin/prednisol ac

349 GFR (glomerular filtration rate)-higher than normal
350 GFR (glomerular filtration rate)-lower than normal
351 GFR (glomerular filtration rate)-normal
352 Glimepiride
353 Glipizide
354 Glomerulonephritis
355 Glucagon,human recombinant
356 Glucose [Mass/volume] in Blood by Automated test strip_abnormal
357 Glucose [mass/volume] in capillary blood by glucometer_abnormal
358 Glucose [mass/volume] in capillary blood_abnormal
359 Glyburide
360 Glyburide,micronized
361 Glyburide/metformin hcl
362 Halobetasol propionate
363 Head vessels Magnetic resonance angiography without contrast
364 Hemoglobin and Hematocrit panel - Blood_abnormal
365 Hemoglobin and hematocrit_abnormal
366 Hemogram_abnormal
367 Hepatitis A virus IgM Ab [Presence] in Serum_abnormal
368 Hepatitis C
369 Hip - bilateral x-ray
370 HIV
371 Hum insulin nph/reg insulin hm
372 Hydralazine
373 hydrochlorothiazide (Hydrodiuril)
374 Hydrocodone bitartrate and acetaminophen
375 Hydrocodone/chlorphen polis
376 Hydrocodone/ibuprofen
377 Hydroxyurea
378 Hyperlipidemia
379 Hypertension
380 Hypertensive diseases
381 Income
382 indapamide (Lozol)
383 Indomethacin
384 Inr in platelet poor plasma by coagulation assay_abnormal
385 Inr_abnormal
386 Insulin aspart
387 Insulin detemir
388 Insulin glargine
389 Insulin isophane
390 Insulin lispro
391 Insulin npl/insulin lispro
392 Insulin regular, human
393 Insuln asp prt/insulin aspart
394 Interferon beta-1b

395 Interstitial nephritis
396 Ipratropium bromide
397 Ipratropium/albuterol sulfate
398 Iron and iron binding capacity panel - serum or plasma_abnormal
399 Iron aspgly&ps cmplx/c/sucac
400 Iron aspgly&ps/c/b12/fa/ca/suc
401 Iron dextran complex (Dexferrum)
402 Iron dextran complex (INFed)
403 Iron Sucrose (Venofer)
404 Isosorbide dinitrate
405 Isosorbide mononitrate
406 Kidney stones
407 Labetalol hcl
408 Lactulose
409 Lamotrigine
410 Lancets
411 Lanthanum carbonate (Fosrenol)
412 Latanoprost
413 Lead exposure
414 Lenalidomide
415 Leukocytes [#/volume] in blood_abnormal
416 Levothyroxine sodium
417 Lidocaine hcl
418 Linagliptin
419 Lisinopril (Zestril and Prinivil)
420 Liver disease
421 Loperamide hcl
422 Losartan potassium
423 lovastatin (Mevacor, Altoprev)
424 Magnesium oxide
425 Manual differential performed [presence] in blood_abnormal
426 Meclizine hcl
427 Medicare beneficiary
428 Melphalan
429 Memantine hcl
430 Mental status changes
431 Meperidine hcl/pf
432 Metabolic syndrome
433 Metformin hcl
434 methyclothiazide (Enduron)
435 metolazone (Zaroxolyn, Diulo, Mykrox)
436 Metoprolol succinate
437 Metoprolol tartrate
438 Mexiletine hcl
439 Micardis
440 Minoxidil

441 Mirtazapine
442 Moexipril (Univasc)
443 Mometasone furoate
444 Morphine sulfate
445 Multivitamin with minerals
446 Muscle cramps
447 Muscle twitches
448 Mycophenolate mofetil
449 Naltrexone hcl
450 Nateglinide
451 Natriuretic peptide B [Mass/volume] in Serum or Plasma_abnormal
452 Natriuretic peptide.B prohormone N-Terminal [Mass/volume] in Serum or Plasma_abnormal
453 Nausea
454 Nifedipine
455 Nitrofurantoin macrocrystal
456 Nitrofurantoin monohydrate/macrocrystals
457 Nitroglycerin
458 Number of outpatient visits
459 Number of pre-admission
460 Nystatin
461 Obesity
462 Occupants per room
463 Olmesartan medoxomil
464 Omega-3-acid ethyl esters
465 Omeprazole
466 Other beneficiary
467 Pantoprazole sodium
468 Parathyroid hormone-higher than normal
469 Parathyroid hormone-lower than normal
470 Parathyroid hormone-normal
471 Paricalcitol (Zemplar)
472 Percent bachelor degree of higher
473 Percent high school graduate of higher
474 Percent low-skilled occupation
475 Percent poverty
476 Percent rural population
477 Percutaneous transluminal venous angioplasty report
478 Perindopril (Aceon)
479 Peripheral vascular disease (PVD)
480 Ph, arterial gas_abnormal
481 Phosphate [mass/volume] in serum or plasma_abnormal
482 pitavastatin (Livalo)
483 Point-of-care testing (POCT) for glucose _abnormal
484 Polycystic kidney disease
485 Polyethylene glycol 3350

486 Potassium [Moles/volume] in Serum or Plasma_abnormal
487 Potassium chloride
488 Potassium-higher than normal
489 Potassium-lower than normal
490 Potassium-normal
491 Pr_9714_replac m/s immob dev nec
492 pravastatin (Pravachol)
493 Prednisone
494 Primary diagnosis_ Burn of unspecified degree of nose (septum)
495 Primary diagnosis_ Intermittent heterotropia, unspecified
496 Primary diagnosis_Acute, but ill-defined, cerebrovascular disease
497 Primary diagnosis_Anemia in end-stage renal disease
498 Primary diagnosis_Anemia unspecified
499 Primary diagnosis_Atrial fibrillation
500 Primary diagnosis_Benign neoplasm of major salivary glands
501 Primary diagnosis_Blister finger
502 Primary diagnosis_Chronic interstitial cystitis
503 Primary diagnosis_Chronic venous hypertension with ulcer (begin 2002)
504 Primary diagnosis_Closed fracture of shaft of fibula with tibia
505 Primary diagnosis_Cong/herid thromb purpra
506 Primary diagnosis_Diabetes with other specified manifestations
507 Primary diagnosis_Dvrtcli colon (w/o hmrhg) (begin 1980)
508 Primary diagnosis_Empyema without mention of fistula
509 Primary diagnosis_Endophthalmitis nec
510 Primary diagnosis_Exotropia, unspecified
511 Primary diagnosis_Giant cell arteritis
512 Primary diagnosis_Heart transplant status
513 Primary diagnosis_Hyper hrt/ren nos w chf (begin 1989)
514 Primary diagnosis_Injury to unspecified blood vessel of thorax
515 Primary diagnosis_Insect bite nec
516 Primary diagnosis_Lesion of ulnar nerve
517 Primary diagnosis_Long-term (current) use of anticoagulants
518 Primary diagnosis_Malig neo subglottis
519 Primary diagnosis_Malignant neo colon, unspecified
520 Primary diagnosis_Malignant neoplasm of bronchus and lung, unspecified
521 Primary diagnosis_Nonhealing surg wnd (begin 1996)
522 Primary diagnosis_Open wnd knee/leg/ankle
523 Primary diagnosis_Other and unspecified hyperlipidemia
524 Primary diagnosis_Other and unspecified intracranial hemorrhage following injury without mention of open intracranial wound, with brief [less than one hour] loss of consciousness
525 Primary diagnosis_Other arterial embolism and thrombosis of abdominal aorta
526 Primary diagnosis_Other diseases of trachea and bronchus
527 Primary diagnosis_Other optic neuritis
528 Primary diagnosis_Other specified antibiotics causing adverse effects in therapeutic use

529 Primary diagnosis_Other specified dermatomycoses
530 Primary diagnosis_Other specified megaloblastic anemias
531 Primary diagnosis_Otorrhea nec
532 Primary diagnosis_Perforation of tympanic membrane, unspecified
533 Primary diagnosis_Peripheral T cell lymphoma, unspecified site, extranodal and solid organ sites
534 Primary diagnosis_Peripheral vascular disease, unspecified
535 Primary diagnosis_Ptosis of eyelid, unspecified
536 Primary diagnosis_Purulent endophthalm nos
537 Primary diagnosis_Secondary malignant neoplasm of mediastinum
538 Primary diagnosis_Senile nuclear sclerosis
539 Primary diagnosis_Spasm of muscle
540 Primary diagnosis_Spontan bact peritonitis (begin 2005)
541 Primary diagnosis_Stridor
542 Primary diagnosis_Subdural hemorrhage following injury without mention of open intracranial wound, unspecified state of consciousness
543 Primary diagnosis_Supraspinatus (muscle) (tendon) sprain
544 Primary diagnosis_Unspecified cirrhosis of liver
545 Primary diagnosis_Varicose veins of lower extremities with ulcer
546 Promethazine hcl
547 Promethazine hcl/codeine
548 Propranolol hcl
549 Protein [mass/volume] in urine_abnormal
550 Protein and Glucose panel - Urine by Test strip_abnormal
551 Protein urine timed collection_abnormal
552 Protein, Urine_abnormal
553 Prothrombin time (pt) in platelet poor plasma by coagulation assay_abnormal
554 Prothrombin time with international normalized ratio
555 PT panel - Platelet poor plasma by Coagulation assay_abnormal
556 Pyelonephritis
557 Quetiapine fumarate
558 Quinapril (Accupril)
559 Race-American Indian and Alaska Native
560 Race-Asian
561 Race-Black or African American
562 Race-Hispanic
563 Race-Native Hawaiian and Other Pacific Islander
564 Race-Other
565 Race-Unknown
566 Race-White
567 Ramipril (Altace)
568 Ranitidine hcl
569 Renal function 2000 panel - serum or plasma_abnormal
570 Renal function panel_abnormal
571 Renter occupied
572 Rifaximin

573 Rivaroxaban
574 Roflumilast
575 Ropinirole hcl
576 rosuvastatin (Crestor)
577 Salmeterol xinafoate
578 Saxagliptin hcl
579 Secondary diagnosis_ Moderate nonproliferative diabetic retinopathy
580 Secondary diagnosis_accidental poisoning by glues and adhesives
581 Secondary diagnosis_Achalasia and cardiospasm
582 Secondary diagnosis_anemia in end-stage renal disease (begin 2000)
583 Secondary diagnosis_atrial fibrillation
584 Secondary diagnosis_Atrophic flaccid tympanic membrane
585 Secondary diagnosis_Attention to ileostomy
586 Secondary diagnosis_autoimmune hepatitis
587 Secondary diagnosis_Bacteroides fragilis
588 Secondary diagnosis_Behcet's syndrome
589 Secondary diagnosis_chronic skin ulcer nec
590 Secondary diagnosis_chronic venous hypertension with ulcer
591 Secondary diagnosis_Congestive heart failure
592 Secondary diagnosis_Dehydration
593 Secondary diagnosis_diabetes mellitus type ii uncontrolled
594 Secondary diagnosis_Diabetes mellitus without mention of complication, type II
595 Secondary diagnosis_Diabetes with neurological manifestations, type I uncontrolled
596 Secondary diagnosis_Diabetes with other specified manifestations
597 Secondary diagnosis_Gastroparesis
598 Secondary diagnosis_Gonococcal peritonitis
599 Secondary diagnosis_Heart transplant status
600 Secondary diagnosis_Hemodialysis
601 Secondary diagnosis_Hypoparathyroidism
602 Secondary diagnosis_Iron deficiency anemia, unspecified
603 Secondary diagnosis_Longterm use anticoag
604 Secondary diagnosis_Malignant neoplasm of body of pancreas
605 Secondary diagnosis_Malignant neoplasm of hypopharynx, unspecified site
606 Secondary diagnosis_Malignant neoplasm of upper respiratory tract, part unspecified
607 Secondary diagnosis_Methicillin susceptible Staphylococcus aureus in conditions classified elsewhere and of unspecified site
608 Secondary diagnosis_myoclonus
609 Secondary diagnosis_Nonexudat macular degeneration
610 Secondary diagnosis_Occlusion and stenosis of multiple and bilateral precerebral arteries without mention of cerebral infarction
611 Secondary diagnosis_Other accident caused by striking against or being struck accidentally by objects or persons
612 Secondary diagnosis_Other and unspecified Escherichia coli
613 Secondary diagnosis_Other and unspecified hyperlipidemia

614 Secondary diagnosis_Other specified anemias
615 Secondary diagnosis_Other specified surgical operations and procedures causing abnormal patient reaction, or later complication, without mention of misadventure at time of operation
616 Secondary diagnosis_other vulvodynia
617 Secondary diagnosis_Partial epilepsy and epileptic syndromes with simple partial seizures
618 Secondary diagnosis_Percutaneous transluminal coronary angioplasty status
619 Secondary diagnosis_Perforation of tympanic membrane, unspecified
620 Secondary diagnosis_rectal prolapse
621 Secondary diagnosis_Reflex sympathetic dystrophy, unspecified
622 Secondary diagnosis_Renal dialysis status
623 Secondary diagnosis_Restless legs syndrome
624 Secondary diagnosis_Secondary malignant neoplasm of lung
625 Secondary diagnosis_Secondary malignant neoplasm of other digestive organs and spleen
626 Secondary diagnosis_stridor
627 Secondary diagnosis_therapeutic drug monitoring
628 Secondary diagnosis_Ulcer of other part of foot
629 Secondary diagnosis_ulcer of other part of lower limb
630 Secondary diagnosis_Unspecified cirrhosis of liver
631 Secondary diagnosis_Unspecified disorder of metabolism
632 Secondary diagnosis_Unspecified heart failure
633 Secondary diagnosis_Unspecified open wound of ocular adnexa
634 Secondary diagnosis_Unspecified protein-calorie malnutrition
635 Secondary diagnosis_urgency of urination
636 Secondary diagnosis_urinary frequency
637 Secondary diagnosis_vitreous degeneration
638 Seizure
639 Serum Albumin-higher than normal
640 Serum Albumin-lower than normal
641 Serum Albumin-normal
642 Serum Bicarbonate-higher than normal
643 Serum Bicarbonate-lower than normal
644 Serum Bicarbonate-normal
645 Serum Calcium-higher than normal
646 Serum Calcium-lower than normal
647 Serum Calcium-normal
648 Serum Creatinine-higher than normal
649 Serum Creatinine-lower than normal
650 Serum Creatinine-normal
651 Serum Phosphorous-higher than normal
652 Serum Phosphorous-lower than normal
653 Serum Phosphorous-normal
654 Sevelamer (renagel)
655 Sevelamer (Renvela)

656 Sevelamer carbonate
657 Sevelamer hcl
658 Shortness of breath
659 Sickle cell trait
660 Sildenafil citrate
661 Silodosin
662 Silver sulfadiazine
663 simvastatin (Zocor)
664 Sitagliptin phosphate
665 Smear review_abnormal
666 Smoking
667 Sodium bicarbonate
668 Sodium polystyrene sulfonate
669 Sotalol hcl
670 Speaking a language other than English
671 Spine cervical CT without contrast
672 Spine cervical x-ray ap and oblique and lateral with flexion and with extension
673 Spine lumbar CT without contrast
674 Spironolactone
675 Sucroferric oxyhydroxide (Velphoro)
676 Sunitinib malate
677 Syring w-ndl,disp,insul,0.5ml
678 Syringe w-ndl, disp., insulin
679 Syringe w-needle,disposab,3ml
680 Tamsulosin hcl
681 Temazepam
682 Terazosin hcl
683 Teveten
684 Theophylline anhydrous
685 Ticlopidine hcl
686 Tiotropium bromide
687 Tolterodine tartrate
688 Topiramate
689 Torsemide
690 Total cost
691 Total length of inpatient stay
692 Total number of chronic conditions
693 Total number of diagnoses
694 Total number of lab tests
695 Total number of lab tests with abnormal results
696 Total number of medications
697 Total number of radiology tests
698 Total Parenteral Nutrition (TPN_abnormal Panel_abnormal
699 Tramadol hcl
700 Trandolapril (Mavik)
701 Triamterene

702	Trimethoprim
703	Troponin i protocol 4_abnormal
704	Type 1 diabetes
705	Type 2 diabetes
706	Unemployment rate
707	Uninsured rate
708	Unspecified hypertension
709	Urea nitrogen [Mass/volume] in Serum or Plasma_abnormal
710	Urinalysis complete w reflex culture panel - urine_abnormal
711	Urinalysis dipstick W Reflex Microscopic panel in Urine_abnormal
712	Urinalysis dipstick with Reflex to Microscopic panel l - urine_abnormal
713	Urinalysis Microscopic_abnormal
714	Urinalysis panel - urine by auto_abnormal
715	Urinalysis reflex testing_abnormal
716	Urinalysis with Microscopic if indicated_abnormal
717	Urinalysis with Microscopic included
718	Urinalysis, culture if indicated_abnormal
719	Urine Albumin Creatinine Ratio-higher than normal
720	Urine Albumin Creatinine Ratio-lower than normal
721	Urine Albumin Creatinine Ratio-normal
722	Ursodiol
723	USP + SEDIMENT (REFLEX)_abnormal
724	Valsartan
725	Valsartan/hydrochlorothiazide
726	Vancomycin hcl
727	Venlafaxine hcl
728	Vesicoureteral reflux
729	Vit b cmplx 3/fa/vit c/biotin
730	VITAMIN D 25 HYDROX_abnormal
731	Vitamin D supplement
732	Vomiting
733	Warfarin sodium
734	Weakness/Fatigue
735	Wrist - right x-ray
736	Zolpidem tartrate
