# Supplementary material for: Estimating One-Year Risk of Incident Chronic Kidney Disease: Retrospective Development and Validation Study Using Electronic Medical Record Data From the State of Maine
Source: JMIR Med Inform. 2017 Jul 26;5(3):e21. doi: 10.2196/medinform.7954 (PMC5550735; doi:10.2196/medinform.7954)
Supplement: Multimedia Appendix 5 [file medinform_v5i3e21_app5.pdf]

Multimedia appendix 5. A list of 146 features (final predictors) and their weights in the model

Differentiating features	Weight
Furosemide	13.20
Age	11.90
Total number of medications	2.77
Chem 6 panel	2.65
Allopurinol	1.98
Unspecified disorders of kidney and ureter	1.84
Race (White)	1.44
Total number of lab tests with abnormal results	1.31
Diabetes mellitus	1.30
Hydralazine	1.30
Congestive heart failure	1.21
Renal disease	1.20
Hypertension	1.00
Medicare beneficiary	0.94
Amlodipine besylate	0.93
Insulin Glargine	0.92
Total number of lab tests	0.75
Outpatient visits	0.70
Bumetanide	0.69
Isosorbide mononitrate	0.62
Income	0.56
Insulin isophane	0.56
Inpatient length of stay	0.56
Glipizide	0.55
Total costs	0.54
Point-of-care testing (POCT) for glucose	0.53
Prothrombin time with international normalized ratio	0.48
Metoprolol tartrate	0.46
Febuxostat	0.43
Insulin Detemir	0.40
Disposable insulin needles	0.35
Valsartan	0.35
Lisinopril	0.34
Prednisone	0.33
Hydrochlorothiazide	0.32
Anemia	0.28
Percent bachelor degree of higher in the community	0.28
Insulin aspart	0.28
Spironolactone	0.27
Atherosclerotic heart disease (ASHD)	0.26

Gender (Male)	0.26
Carvedilol	0.26
Amiodarone	0.25
Colchicine	0.24
Total number of diagnoses	0.23
Therapeutic drug monitoring	0.23
Potassium chloride	0.23
Total number of radiology tests	0.23
Disorders of lipid metabolism	0.22
Folic acid	0.21
Dexlansoprazole	0.21
Atenolol	0.21
Atorvastatin calcium	0.19
Terazosin HCL	0.19
Rosuvastatin calcium	0.18
Glyburide	0.18
Clopidogrel bisulfate	0.17
Albuterol sulfate	0.16
Long term use anticoag	0.16
Hyperlipidemia	0.15
Insulin lispro	0.15
Diabetes mellitus without mention of complication, type II or unspecified type, uncontrolled	0.15
Dabigatran etexilate mesylate	0.14
Warfarin sodium	0.14
Urinalysis with Microscopic if indicated	0.14
Azithromycin	0.14
Fenofibrate nanocrystallized	0.14
Hydrocodone Bitartrate and Acetaminophen	0.13
Losartan potassium	0.13
Hum Insulin Nph/Reg Insulin Hm	0.12
Levothyroxine sodium	0.12
Simvastatin	0.12
Blood sugar diagnostic	0.11
Cephalexin	0.10
Tamsulosin HCL	0.10
Glucose [Mass/volume] in Capillary blood	0.10
Edema	0.09
Digoxin	0.09
Tramadol HCL	0.08
Nitrofurantoin monohydrate/macrocrystals	0.08
Coronary atherosclerosis and other heart disease	0.08
Zolpidem tartrate	0.08
CBC with Differential	0.08
Commercial health insurance beneficiary	0.08

Acyclovir	0.07
Budesonide/formoterol fumarate	0.07
Finasteride	0.07
Gabapentin	0.07
Metoprolol succinate	0.07
Occlusion or stenosis of precerebral arteries	0.07
Dysrhythmia	0.07
Non exudative macular degeneration	0.07
Glimepiride	0.07
Ranitidine HCL	0.07
Activated partial thromboplastin time (APTT) in platelet poor plasma by coagulation assay	0.06
Amitriptyline HCL	0.06
Vitreous degeneration	0.06
Latanoprost	0.06
Venlafaxine HCL	0.05
Pantoprazole sodium	0.05
Nitroglycerin	0.05
Omeprazole	0.05
Ropinirole HCL	0.05
CBC with auto differential panel in blood	0.05
Type II diabetes	0.05
Urinalysis with Microscopic included	0.05
Tiotropium bromide	0.05
Peripheral and visceral atherosclerosis	0.05
Bimatoprost	0.05
Basic metabolic 2000 panel - Serum or Plasma	0.04
Citalopram hydrobromide	0.04
Other nutritional; endocrine; and metabolic disorders	0.04
Fluticasone propionate	0.04
Famotidine	0.04
1 mL Insulin Syringes with Needle	0.04
Chronic obstructive pulmonary disease and bronchiectasis	0.04
Unspecified anemia	0.04
Atrial fibrillation	0.04
Other liver diseases	0.04
Nutritional deficiencies	0.04
Unspecified essential hypertension	0.04
Clotrimazole and betamethasone dipropionate	0.04
Cerebrovascular accident (CVA) / transient ischemic attack (TIA)	0.04
Nitrofurantoin macrocrystal	0.03
Sitagliptin phosphate	0.03
Cardiac, other	0.03
Type I diabetes	0.03

Fluticasone/salmeterol	0.03
Ezetimibe	0.03
Hemogram test	0.03
Race (Unknown)	0.03
Smear review	0.03
Ulcer of other part of lower limb	0.03
Other and ill-defined heart disease	0.02
Complication of device; implant or graft	0.02
Comprehensive metabolic panel	0.02
Aortic; peripheral; and visceral artery aneurysms	0.02
Nystatin	0.02
Creatinine panel	0.02
Diverticulitis of colon (without mention of hemorrhage)	0.02
Chronic ulcer of skin	0.02
Pravastatin sodium	0.02
Syring W-Ndl,Disp,Insul,0.5Ml	0.02
Urinalysis Reflex testing	0.02
Omega-3-Acid Ethyl Esters	0.02
Urinalysis dipstick with Reflex to Microscopic panel - Urine	0.02
