# Supplementary material for: Estimating One-Year Risk of Incident Chronic Kidney Disease: Retrospective Development and Validation Study Using Electronic Medical Record Data From the State of Maine
Source: JMIR Med Inform. 2017 Jul 26;5(3):e21. doi: 10.2196/medinform.7954 (PMC5550735; doi:10.2196/medinform.7954)
Supplement: Multimedia Appendix 6 [file medinform_v5i3e21_app6.pdf]

Multimedia appendix 6. Top 50 features (i.e. predictors) in the final model: weight, log odds ratio (OR), and 0.95 confidence interval (CI)

**Category**	**Differentiating features**	**Weight**	**Log OR (0.95 CI)**
Demographics	Age	11.9	15.2 (8.4-31.2)
	Race (white)	1.44	4.4 (2.1-4.6)
	Medicare beneficiary	0.94	2.0 (1.7-2.3)
	Sex (male)	0.26	2.1 (1.1-3.8)
Social economic covariates	Income	0.56	0.5 (0.2-1.4)
	Education level	0.28	0.9 (0.6-1.4)
Diagnosis	Unspecified disorders of kidney and ureter	1.84	73.7 (3.6-588.1)
	Diabetes mellitus	1.30	26.2 (12.8-50.6)
	Congestive heart failure	1.21	21.4 (9.1-44.8)
	Acute or unspecified renal failure	1.20	17.7 (1.1-97.7)
	Hypertensive disease	1.00	9.1 (5.1-16.4)
	Anemia	0.28	12.0 (5.9-22.8)
	Atherosclerotic heart disease	0.26	14.1 (7.1-26.4)
	Total number of diagnoses	0.23	2.0 (1.7-2.4)
	Disorders of lipid metabolism	0.22	9.0 (5.0-16.3)
Medication	Furosemide[a]	13.2	2.1 (1.7-2.5)
	Allopurinol	1.98	2.1 (1.5-2.7)
	Hydralazine[b]	1.30	2.1 (0.8-3.5)
	Amlodipine besylate[b]	0.93	1.8 (1.4-2.2)
	Insulin Glargine[c]	0.92	1.5 (0.8-2.1)
	Bumetanide[b]	0.69	2.0 (0.9-3.1)
	Isosorbide mononitrate	0.62	1.3 (0.4-2.1)
	Insulin isophane[c]	0.56	2.5 (1.6-3.8)
	Glipizide[c]	0.55	1.4 (0.6-2.1)
	Metoprolol tartrate[b]	0.46	1.7 (1.3-2.1)
	Febuxostat	0.43	7.3 (2.4-43.4)
	Insulin Detemir[c]	0.40	1.8 (0.9-2.7)
	Disposable insulin needles[c]	0.35	2.0 (1.0-2.9)
	Valsartan[a,b]	0.35	1.9 (1.0-2.8)
	Lisinopril[a,b]	0.34	1.8 (1.5-2.1)
	Prednisone	0.33	1.8 (1.4-2.3)
	Hydrochlorothiazide[b]	0.32	1.3 (0.9-1.8)
	Insulin aspart[c]	0.28	1.6 (0.8-2.3)
	Spironolactone[b]	0.27	2.5 (1.7-3.3)
	Carvedilol[a,b]	0.26	1.5 (0.6-2.4)
	Amiodarone[a]	0.25	3.3 (0.7-7.8)
	Colchicine	0.24	2.8 (1.0-5.1)
	Potassium chloride	0.23	2.0 (1.6-2.5)
	Folic acid	0.21	1.5 (0.5-2.5)
Laboratory test	Chem 6 panel	2.65	1.6 (1.4-1.8)
	Point-of-care testing for glucose	0.53	1.6 (1.0-2.1)

	Prothrombin time with international normalized ratio	0.48	1.7 (1.3-2.1)
	Therapeutic drug monitoring	0.23	2.4 (0.6-5.0)
Utilization	Total number of medications	2.77	2.0 (1.7-2.3)
	Total number of laboratory tests with abnormal results	1.31	1.6 (1.4-1.8)
	Total number of laboratory tests	0.75	1.4 (1.3-1.6)
	Outpatient visits	0.70	1.9 (1.5-2.2)
	Inpatient length of stay	0.56	1.9 (1.5-2.3)
	Total costs	0.54	1.2 (1.1-1.3)
	Total number of radiology tests	0.23	1.2 (1.0-1.4)

[a]Heart disease medication

[b]Blood medication

[c]Diabetes medication
