# Supplementary material for: Estimating One-Year Risk of Incident Chronic Kidney Disease: Retrospective Development and Validation Study Using Electronic Medical Record Data From the State of Maine
Source: JMIR Med Inform. 2017 Jul 26;5(3):e21. doi: 10.2196/medinform.7954 (PMC5550735; doi:10.2196/medinform.7954)
Supplement: Multimedia Appendix 7 [file medinform_v5i3e21_app7.pdf]

Multimedia appendix 7. A two-by-two contingency table for the derivation cohort and the validation cohort. The cutoff score is 0.05.

	Derivation		Validation	
	**CKD (–)**	**CKD (+)**	**CKD (–)**	**CKD (+)**
**Predicted as CKD (–)**	1,268,143	2785	1,374,056	4122
**Predicted as CKD (+)**	34,772	4663	48,417	4177
