# Supplementary material for: Estimating One-Year Risk of Incident Chronic Kidney Disease: Retrospective Development and Validation Study Using Electronic Medical Record Data From the State of Maine
Source: JMIR Med Inform. 2017 Jul 26;5(3):e21. doi: 10.2196/medinform.7954 (PMC5550735; doi:10.2196/medinform.7954)
Supplement: Multimedia Appendix 8 [file medinform_v5i3e21_app8.pdf]

Multimedia appendix 8. Relationships between sensitivities, specificities, and PPVs of the model on the validation cohort

Sensitivity, %	Specificity, %	PPV, %
80.33	87.88	3.72
75.43	90.54	4.45
70.50	92.51	5.21
65.51	93.82	5.83
60.44	94.92	6.49
55.25	95.84	7.19
50.33	96.60	7.94
