# Supplementary material for: Estimating One-Year Risk of Incident Chronic Kidney Disease: Retrospective Development and Validation Study Using Electronic Medical Record Data From the State of Maine
Source: JMIR Med Inform. 2017 Jul 26;5(3):e21. doi: 10.2196/medinform.7954 (PMC5550735; doi:10.2196/medinform.7954)
Supplement: Multimedia Appendix 9 [file medinform_v5i3e21_app9.pdf]

Multimedia appendix 9. Comparison of c-statistics using other clinical prediction scores

**Model**	**Cohort**	**Predictors**	**c-statistic**
Hemmelgarn et al, 2007[1]	Patients aged 66+ and have serum creatinine measurements (n=6789 in derivation and n=3395 in validation)	Age, heart diseases, diabetes, gout, anti-emetic medications	0.59
Thakkinstian et al, 2011[2]	Patients aged 18+ (n=3459 for derivation)	Age, diabetes, hypertension, and history of kidney stones	0.741
O'Seaghdha et al, 2012[3]	Patients from Framingham Heart Study (n=2490) for derivation and ARIC study (n=1777) for validation, and have serum creatinine measurements	Age, diabetes, hypertension, eGFR, albuminuria	0.813
Our model	Patients from Maine HIE databases (n=1,310,363 for derivation and n=1,430,772 for validation)	146 clinical variables including demographics, diagnosis, medications, laboratory test results, and resource utilization	0.871

1. Hemmelgarn, BR, Culleton, BF, Ghali, WA: Derivation and validation of a clinical index for prediction of rapid progression of kidney dysfunction. *QJM : monthly journal of the Association of Physicians*, 100**:** 87-92, 2007.
2. Thakkinstian, A, Ingsathit, A, Chaiprasert, A, Rattanasiri, S, Sangthawan, P, Gojaseni, P, Kiattisunthorn, K, Ongaiyooth, L, Thirakhupt, P: A simplified clinical prediction score of chronic kidney disease: a cross-sectional-survey study. *BMC Nephrol*, 12**:** 45, 2011.
3. O'Seaghdha, CM, Lyass, A, Massaro, JM, Meigs, JB, Coresh, J, D'Agostino, RB, Sr., Astor, BC, Fox, CS: A risk score for chronic kidney disease in the general population. *Am J Med*, 125**:** 270-277, 2012.
